# Inferring multi-locus selection in admixed populations

**DOI:** 10.1371/journal.pgen.1011062

**Published:** 2023-11-28

**Authors:** Nicolas M. Ayala, Maximilian Genetti, Russell Corbett-Detig

**Affiliations:** 1 Genomics Institute, University of California, Santa Cruz; Santa Cruz, California, United States of America; 2 Department of Biomolecular Engineering, University of California, Santa Cruz; Santa Cruz, California, United States of America; CNRS UMR5554, FRANCE

## Abstract

Admixture, the exchange of genetic information between distinct source populations, is thought to be a major source of adaptive genetic variation. Unlike mutation events, which periodically generate single alleles, admixture can introduce many selected alleles simultaneously. As such, the effects of linkage between selected alleles may be especially pronounced in admixed populations. However, existing tools for identifying selected mutations within admixed populations only account for selection at a single site, overlooking phenomena such as linkage among proximal selected alleles. Here, we develop and extensively validate a method for identifying and quantifying the individual effects of multiple linked selected sites on a chromosome in admixed populations. Our approach numerically calculates the expected local ancestry landscape in an admixed population for a given multi-locus selection model, and then maximizes the likelihood of the model. After applying this method to admixed populations of *Drosophila melanogaster* and *Passer italiae*, we found that the impacts between linked sites may be an important contributor to natural selection in admixed populations. Furthermore, for the situations we considered, the selection coefficients and number of selected sites are overestimated in analyses that do not consider the effects of linkage among selected sites. Our results imply that linkage among selected sites may be an important evolutionary force in admixed populations. This tool provides a powerful generalized method to investigate these crucial phenomena in diverse populations.

## Author summary

In this text we introduce a method for modeling the transition rates between local ancestry states at sites along a genome which may be in linkage to multiple sites under selection. We implement our method as a tool called AHMM_MLS which we validate with simulations and apply to admixed systems of *Drosophila melanogaster* and *Passer italiae*. In our validations we found that our tool can accurately detect the number of sites and predict their selection coefficients even when they are in linkage. We also found that the inferred selection coefficients of selected sites in linkage may be overestimated if the effects of linkage are ignored. In applying our tool to two admixed systems, we found that our lab’s previous tool AHMM-S may overestimate the number and selection coefficients of selected positions along chromosomes in these populations. Our multi-locus selection model for these admixed chromosomes produces an expected local ancestry that closely matches the local ancestry inferred from samples. We expect that our tool will allow greater insights into admixed chromosomes which show signs of a few strong selected positions that are in linkage.

## Introduction

Admixture is one of the primary sources of selected alleles in natural populations [1–3]. For example, in *Helianthus* sunflowers, introgressed alleles enhanced herbivore resistance at a number of loci [4]. In the fish *Fundulus grandis*, recently introgressed alleles allow resistance to extreme pollution and environmental change [5]. In humans, introgression from archaic hominids is thought to have facilitated adaptation to a range of novel environments [6,7]. Similarly, admixture can contribute alleles that are not adapted to local environments (*e*.*g*., as in [8]) or it may contribute haplotypes that are deleterious due to accumulation of weakly deleterious mutations during long term isolation in small populations [9], and are therefore purged by natural selection. Finally, in some cases admixed populations may contain mutations contributed by separate parental populations that have negative interactions thereby resulting in strong selection within the admixed populations [8,10]. Although the importance of selection on admixed ancestry, or adaptive introgression, is increasingly appreciated, generalized methods to accurately detect and quantify the impacts of natural selection from genome sequence data in admixed populations are in their infancy.

Admixture may be disproportionately likely to create circumstances where selected sites affect the evolutionary dynamics of other selected sites through linkage. The effects of multi-locus (non-epistatic) selection have been studied extensively in the context of populations along a geographic cline. Theory demonstrates that closely linked locally selected alleles can strongly reshape their expected frequencies across geographic clines (*e*.*g*., [11–13]), and fixation probabilities in a continent-island model (*e*.*g*., [14–17]). More generally, linkage among selected alleles can generate dynamics in clines that include combinations of loci involved in complex selection (*e*.*g*., [18]). Although multi-locus selection in admixed populations generated through a single admixture event, sometimes called an “admixture pulse”, has been less extensively studied (although see *e*.*g*., [9]), linkage among selected alleles should also impact evolutionary dynamics in such admixed populations. For example, if each ancestral population contributes distinct adaptive variants that are closely linked, their fixation could be impeded by Hill-Robertson interference [19,20]. Conversely, and of particular relevance to our applications below, if a single population contributes linked adaptive variants, their collective allele frequency change could exceed expectations for a single locus due to complementary hitchhiking effects. For example, we might expect the latter in circumstances where one ancestral population has recently undergone polygenic adaptation for a trait that remains beneficial within the admixed population. It is therefore important to develop inference methods for detecting and quantifying the impacts of multiple selected alleles within admixed populations.

General frameworks for detecting selection within admixed populations have developed substantially in recent years, but they suffer from issues that affect their precision and applicability, and so far, none have addressed the challenges of accurate inference with multiple linked selected alleles. Many applications search for increases in allele frequencies at sampled sites, but these sites have to be known in advance in order to be sampled [21–23]. Other applications search for outliers in average local ancestry—the ancestry of admixed individuals at particular loci—after applying tools that assume a neutral and uniform admixture process [24–34]. However, selection in an admixed population itself shapes the landscape of local ancestry, and most tools do not incorporate information about the ancestry tract length distribution. The ancestry tracts, or contiguous portions of the genome that are inherited from a single ancestral population, can reveal portions of the genome which have hitchhiked due to selected loci. Other approaches have been developed that use summary statistics to detect selection acting in admixed populations, but most do not provide a means to estimate the selection coefficients of the sites under selection [35–39]. Machine learning approaches can be quite powerful for detecting adaptive introgression, but they also usually do not provide a means to estimate selection coefficients, and it is sometimes difficult to interpret the biological underpinnings of the model [40–42]. Our lab’s recently developed method resolves some of these difficulties by explicitly modeling selection during admixture in sequence alignment data rather than genotypes as a part of local ancestry inference [43]. Using this approach, it is possible to fit a model with a single locus experiencing additive selection [43]. Although a huge range of new methods are rapidly being developed, thus far, none have considered the effects of multiple linked selected sites.

The previous approaches are suitable for finding evidence of selection at a single site in an otherwise neutrally-evolving genome, but in general they do not account for cases where multiple selected sites are genetically linked and may affect the trajectories of each other due to linkage [19,20,43]. Existing methods are expected to incorrectly estimate the selection coefficients of individual sites when they are impacted by linkage with other selected alleles. This makes estimating the selection coefficient of each variant more difficult within admixed populations [43]. These methods also cannot distinguish between single and multiple site selection models, which may lead to an overestimation of the number of selected sites present on a chromosome within an admixed population in some circumstances. We therefore do not have the tools to investigate the impacts of multi-locus selection in admixed populations, but we expect that this phenomenon is widespread for the reasons we described above.

We introduce an approach for modeling the effects of linkage between multiple selected sites within admixed populations of eukaryotic sexually recombining species. We validated our method under a variety of simulated scenarios, where the introgressing population introduced multiple alleles under selection. This approach can accurately identify the number of linked selected sites, as well as determine their location and estimate their selection coefficients by considering the impacts of the linked selected alleles. We applied our method to an admixed population of *D*. *melanogaster*, and we show that our previous method may have overestimated both the number of selected sites and their selection coefficients due to the effects of linkage [43]. We also applied our method to an admixed population of *P*. *italiae*, and similarly found that fitting single site models may overestimate the selection coefficients of selected positions when compared to multi-locus models. Our results suggest that this is an important contributor to evolutionary outcomes in admixed populations, and our work provides a powerful generalized tool to quantitatively investigate these effects.

## Description of the method

### Model overview

To investigate the impacts of selection on many linked sites, we developed Ancestry-HMM Multi-Locus-Selection (AHMM-MLS). AHMM-MLS is an extension of Ancestry_HMM [44], the latter of which infers both local ancestry and time since admixture for admixed populations by modeling local ancestry in a set of samples from the admixed population as a hidden Markov model (HMM) using a neutral single or multi-pulse admixture model [45]. Our framework considers only single pulse admixture demographic models, where admixture occurs *t* generations prior to sampling. The hidden states of the HMM are the local ancestries of the samples at ancestry informative positions—*i*.*e*. the positions sampled from along the genome in genotype or pileup data whose allele frequencies differ between the ancestral populations—and the observed states are the alleles in the aligned reads at these positions. The emission probabilities of the HMM are computed based on the read alignment data from admixed samples and the allele frequencies in reference unadmixed populations (see [44] for details). AHMM-MLS uses the same emission probabilities as in prior work [44].

Our method introduces a new technique to infer the expected transition probabilities between ancestry types at adjacent ancestry informative sites along the chromosome under generalized models of multi-locus selection during admixture in a single pulse model (see below). Because alleles near selected sites tend to hitchhike to the selected site, the tract lengths for contiguous regions of local ancestry tend to be longer around selected sites [46], and this effect can be captured in the transition probabilities between ancestry types. We compute the expected transition probabilities using a numerical method (see below) and then use the forward algorithm to compute model likelihoods given the samples [47], and a direct search algorithm to optimize the multi-locus selection model. We do not consider epistatic interactions among sites in this framework, but we consider their effects on selection inference (see Verification and Comparison). Our approach also does not include the effects of genetic drift and instead assumes the admixed population is infinite. Below we evaluated the impacts of population size on resulting inferences (see Verification and Comparison), but as an approximation we expect that drift will have small effects in large or moderate admixed populations (S1 Fig).

### Generating transition probabilities

To calculate the effects of multiple selected sites on the expected ancestry transition probabilities between two adjacent ancestry informative sites, we use a numerical method to track the expected distribution of haplotypes after *t* generations of admixture, including the local ancestries of the selected sites and the two ancestry informative sites. If there are *n* selected sites, then we track *n* + 2 total positions (i.e., selected positions and ancestry informative sites), leading to 2^*n*+2^ haplotypes. We do not assume the selected sites are sampled as ancestry informative markers in the admixed population and their positions may lie anywhere along the chromosome. The haplotype distribution in the admixed population at generation 0 is modeled by a row vector *H*^0^, which undergoes a transformation in each generation, producing a sequence of vectors *H*^0^…*H*^*t*^, one for each generation. The vector *H*^*g*^ is transformed into the vector *H*^*g*+1^, representing the expected change in the haplotype distribution from generation *g* to *g +* 1. As a convention for this work, *H*^*g*^_0_ is the frequency of the haplotype consisting of positions all originating from ancestral population 0, (i.e. the ancestral origin of the sites along the chromosome is 00…00), and *H*^*g*^_1_ is the frequency of the haplotype 00…01, and *H*^*g*^_2_ of haplotype 00…10, and so on, counting in a binary fashion. At generation 0, only two haplotypes, *H*^0^_0_ and ${H^{0}}_{2^{n+2}-1}$, have non-zero frequencies. That is because we assume a single admixture pulse which begins from unadmixed population founders *t* generations before the time of sampling. These are the two haplotypes where all sites are of the same ancestry, and their initial values are dictated by the admixture fraction *m*.


$${H^{0}}_{0}=m$$

$${H^{0}}_{2^{n+2}-1}=1-m$$

$${H^{0}}_{i}=0\;if\;i\neq 0\;and\;i\neq 2^{n+2}-1$$

In each generation, we take the tensor product of *H*^*g*^ with itself to produce a row vector, *D*. This vector corresponds to the expected distribution of diploid individuals that result from the haplotypes assuming an infinite population, random mating, and no segregation distortion.


$${D^{g}}_{i*2^{n+2}+j}={H^{g}}_{i}*{H^{g}}_{j}$$

**Fig 1:**
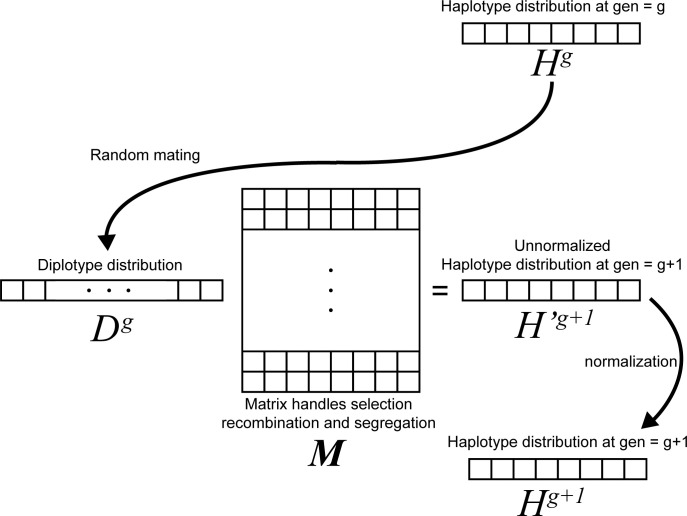
Single generation process for updating haplotype distribution vector. At generation 0, the haplotype vector *H*^*0*^ is initialized to only have non admixed haplotypes, and the matrix ***M*** is computed. In each generation, the diploid genotype distribution *D* is computed from the haplotype distribution by assuming random mating. When the ***M*** operation is applied to *D*, it results in the unnormalized haplotype distribution of the next generation, which is then normalized.

Now, to go from this diploid genotype distribution to the haplotype distribution of the next generation, we apply the matrix operation ***M*** to *D*^*g*^, the result of which is *H*′^*g*+1^. *H*′^*g*+1^ is then normalized to produce *H*^*g*+1^ (Fig 1).


$${H\prime }^{g+1}=D^{g}M$$

$${H^{g+1}}_{j}=\frac{{{H\prime }^{g+1}}_{j}}{\sum_{i=0}^{2^{n+2}}{{H\prime }^{g+1}}_{i}}$$

We call the matrix ***M*** the diploid to haploid transformation, as it converts the diploid genotype distributions of one generation to the expected haplotype distribution of the next generation, accounting for the effects of recombination and natural selection within diploid individuals. A specific entry, such as ***M***_*i*,*j*_, is the contribution of the diploid genotype *i* on the haplotype *j*, taking into account the fitness of the diploid genotype *i*, and the probability that a recombination event produces the haplotype *j*. The matrix depends on the location of the two adjacent ancestry informative sites, the location of every selected site, and the fitness coefficients of those selected sites. Therefore, ***M*** needs to be recomputed for each pair of adjacent ancestry informative sites, and each multi-locus model, but remains constant for each generation of the forward computation. Each selected position is determined by its position on the chromosome in Morgans, and two fitness coefficients. The two fitness coefficients are the dominance coefficient, *h*, and the selection coefficient, *s*. This would make the relative selection coefficients 1, 1—*hs*, 1—*s*. To simplify calculations, we assume all ancestry informative sites are neutral sites where *s* = 0. Using this procedure we can compute transition rates for around 30,000 sampled sites in a matter of seconds, making this method ideal for genome-wide data.

To generate ***M***, we iterate through all possible diploid genotypes. We assume that fitness values combine across selected sites multiplicatively. For a particular diploid genotype *i*, it has an associated fitness *S*_*i*_, which we compute by taking the product of the relevant selection coefficients for each site. For this genotype *i*, we iterate through each region where a recombination event may occur. If there are *n* sites, then there are *n +* 1 regions. Each of these regions has a corresponding recombination rate *r*. Recombination in a specific region would produce two haplotypes, *k* and *l*. So for each recombination event of this genotype, there would be a contribution proportional to *D*_i_*S_i_*r to haplotypes *k* and *l*. This contribution is reflected in ***M*** by adding *S*_i_*r to the ***M***_*i*,*k*_ and ***M***_*i*,*l*_ entries. ***M*** is computed after we have iterated through all possible diploid genotypes and added their contributions to each haploid that they may produce through meiosis. By reducing the most computationally expensive parts of the numerical procedure to matrix multiplications, we are able to use the optimized linear algebra library armadillo [48,49] to quickly compute the transition probabilities for each pair of adjacent ancestry informative sites.

After iterating for *t* generations, we are left with the haplotype distribution that is expected under our model of selection assuming an infinite admixed population size. We directly calculate the transition probabilities between the local ancestries of the ancestry informative sites by iterating through the haplotype distribution and recording the rates of the four possible ancestry state combinations for the two ancestry informative sites.

### Model optimization

Once we have the expected transition probabilities for a particular model, we use the forward algorithm to compute the likelihood of this model given the read pile-up or genotype data [47]. We optimize the parameters of the model to maximize its likelihood using the Nelder-Mead search simplex optimization algorithm [50]. For our hyperparameter values we used a reflection constant of 1, a contraction constant of 0.5, an expansion constant of 2, and a shrinkage constant of 0.5. For each site optimized with unrestricted selection, we optimize the location of the site and two selection coefficients, *h* and *s*. If *h* is known and fixed (*e*.*g*., if additive *h* = 0.5), then only one selection parameter needs to be optimized.

For each optimization, an initial starting point for the location of the selected sites must be supplied by the user. Each optimization is done in two stages, where in the first stage the simplex is centered around the supplied starting point, while in the second stage the simplex is centered around the optimum of the first stage. Each stage consists of multiple starts, with different simplex sizes and orientations. If the range of log likelihoods for every point in the simplex falls below a certain threshold, or if four shrinkage transformations occur in a row, then that search is stopped and the optimum of the simplex is taken to be the optimum of that search. In the first stage, the search is stopped if the range falls below 5 and it falls below a quarter of the initial simplex range. In the second stage, the search is stopped if the range falls below 0.1 and it falls below one 20th of the initial simplex range.

### An iterative multi-locus model selection procedure

**Fig 2:**
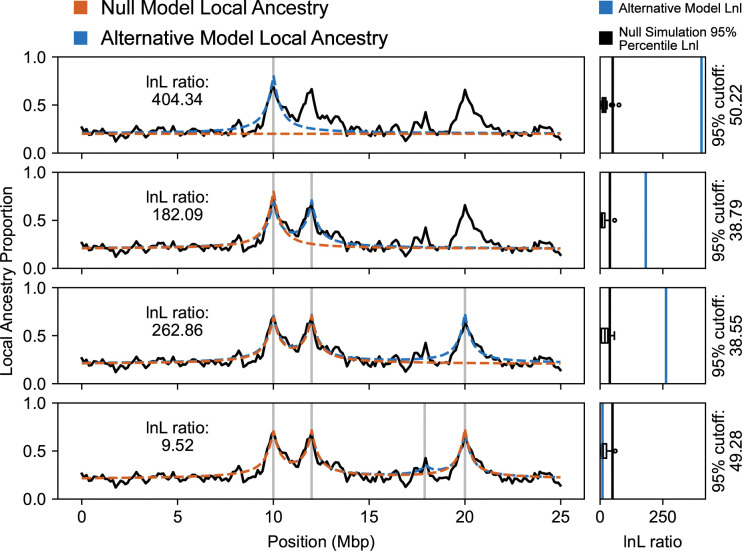
An example of our model selection procedure with four candidate selected positions. On the left, each panel shows the estimated local ancestry proportion within the admixed population (black). On the right, each panel shows the distribution of log likelihood ratios between the alternative and the null model on simulations of the null model (black), and on the underlying data (blue). In the first iteration (top), we test a single site selection model (blue) against a neutral null model (orange). We then test a two-site selection model (blue, second row) against the previous single site selection model which is now the null model (orange, second row). In the third iteration, we test a three-site selection model (blue, third row) against the two-site selection model from the second iteration (orange, third row). Finally, in testing a four-site selection model (blue, bottom), the likelihood ratio (blue, bottom right) does not exceed the 95th percentile of null simulations (black line, bottom right) of the previously selected three-site model (orange, bottom). The method terminates and accepts the three-site selection model obtained in the third iteration. In each panel, vertical gray lines indicate the positions of selected sites in the alternative model considered.

We developed, tested, and implemented an iterative procedure for fitting a multi-locus selection model to genotype data from admixed populations (Fig 2). Our method begins by identifying a set of potential selected sites using Ancestry_HMM-S [43], and applying basic local-optimum selection approaches to remove trivially close positions that may correspond to a single selected allele. We expect that even basic local-ancestry outlier analysis [51,52] might be sufficient for generating a set of candidate positions, but we do not evaluate alternatives here. We then sort the list of candidate selected sites by decreasing likelihood ratio. In the first iteration, we test the site with the highest likelihood ratio as a single site selection model against a null model consisting of a neutral admixture with similar demographic parameters. In each subsequent iteration, we add one additional selected position, constructing an alternative model that we test against the model obtained from the previous iteration.

Our method makes simplifying assumptions about the admixed and ancestral populations that may make it unsuitable for common model selection methods such as the Bayesian information criterion or the Akaike information criterion (S2 Fig) [53,54]. Instead, to obtain the expected distribution of likelihood ratios under the null model, we perform simulations of populations with selected positions that match this model, and we simulate the sampling of reads from these populations. Simulations under the null model show that population size is a contributor to additional variation not captured by the theoretically expected distribution (S2 Fig), and we expect that other unmodelled components may also affect inferences. On these simulated reads expected from the null model, we fit both the null model, and an alternative model which includes the same sites as the null but additionally includes the next site in our sorted list of candidate selected positions, and we note the likelihood ratio between these two estimated models. Essentially, evidence for selection at each site is evaluated in the context of all previously estimated selected sites. When an alternative model exceeds the 95th percentile of the simulated null model likelihood ratio distribution, we accept that position and this new multi-locus selection model becomes the null model in the next iteration. If a given position does not exceed the significance threshold, we discard it and attempt the same procedure with the next candidate position against the same null model. The procedure terminates once we have either accepted or rejected every candidate selected site (Fig 2). We use this same technique of simulating populations to obtain a null distribution for all of our verifications and applications below.

## Verification and comparison

### Model validation with forward simulations

To evaluate our method over a large variety of plausible introgression scenarios, we performed forward simulations. In each of our simulations, a diploid population received a single admixture pulse from another population carrying at least one selected allele, producing a large admixed population (*n* = 10000, unless stated otherwise). As in prior work [43–45], we first used the coalescent simulation program MaCS [55] to create the genotype data for unadmixed individuals. We simulated the local ancestry along the genome of admixed samples using SELAM [56]. This procedure is described in detail in prior work [44,45]. For each population and selection scenario, we simulated 20 admixture events that aligned with the null model and 20 that aligned with the alternative model. We used GNU parallel to run many batches of simulations at once [57]. We sampled 75 diploid individuals from each of these simulations, except in the simulations of the *D*. *melanogaster* and *P*. *italiae* populations below, where we sample the same number of individuals from our simulation as we have sampled from the population, which were 44 and 31 respectively.

**Fig 3:**
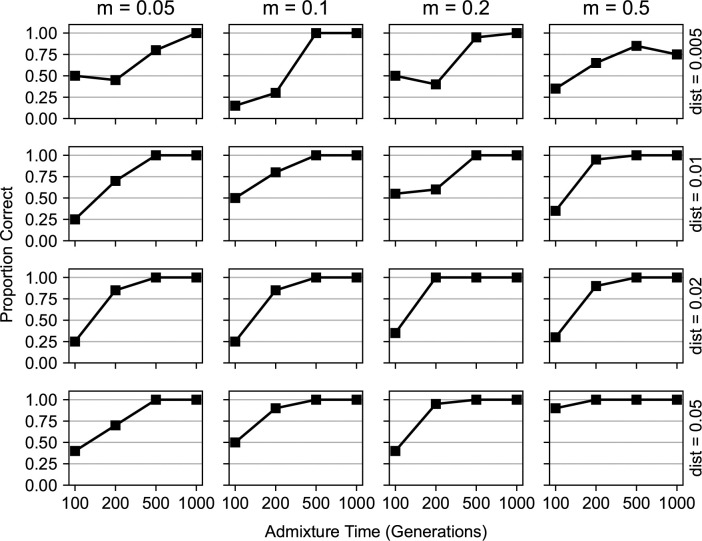
Performance of our method in detecting two nearby selected sites. We evaluated AHMM-MLS in its ability to distinguish between the presence of two nearby sites under selection and the presence of a single site under selection. We ran simulations with varying minor ancestry fractions (0.05, 0.1, 0.2, and 0.5 from left to right), times since admixture (100–1000 generations), and distances between selected positions (0.005, 0.01, 0.02 and 0.05 Morgans, from top to bottom). In each simulation, the selection coefficient of both sites was 0.01. There were a total of 64 different combinations of demographic and selection model parameters. We also ran null model simulations, where there was only a single site under selection introduced in the admixture event, to establish a null model distribution. The points on the lines indicate the proportion of two site simulations in which the single site null model was correctly rejected.

In evaluating performance with simulated data, we found that our method could accurately distinguish between a single selected site and two nearby selected sites over varying population parameters (Fig 3). We varied the time since admixture *t* from 100 to 1000 generations, the admixture proportion from 0.05 to 0.5, and the distance in Morgans between the selected sites from 0.005 to 0.05. We found that one and two site selection models are easier to distinguish as *t* increases, and as the distance between the sites increases. This is because the selected positions have more time to rise in allele frequency and be broken up into separate haplotypes. Our method could also accurately predict the location of the two nearby sites (S3 Fig), when the number of generations is high enough (*t* ⪆ 500). The accuracy of the predicted locations was not affected by the distance between the sites (S3 Fig). In most cases, our method performed better as the time since admixture increased, and as the strength of selection increased.

**Fig 4:**
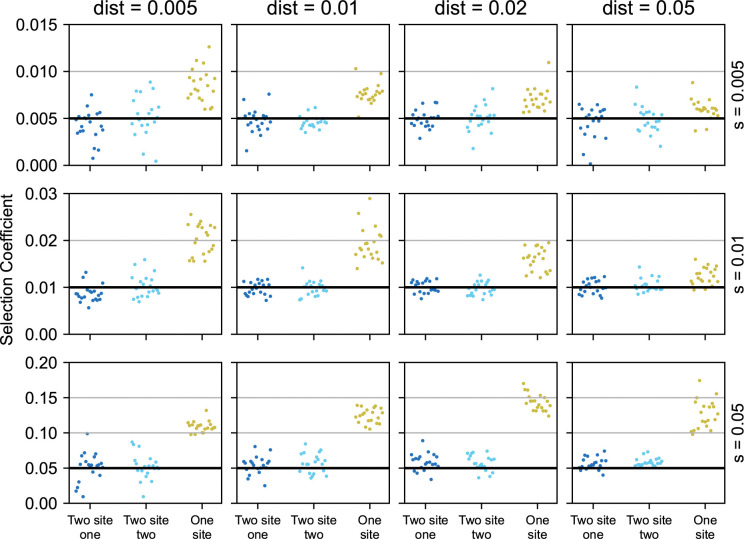
Comparing the inferred selection coefficients between single site and two site models. We evaluated the estimated selection coefficients when using two different models to approach the same data. We ran simulations with varying selection coefficients (0.005, 0.01, and 0.05 from top to bottom) and distances between selected positions (0.005, 0.01, 0.02 and 0.05 Morgans, from left to right). On each simulation we fit a two-site model and a single site model. The dark blue and the light blue dots indicate the inferred selection coefficients of the two sites in the two-site model, while the yellow dots are the inferred selection coefficients of the single site model. The horizontal black lines indicate the simulated selection coefficients.

We found that optimizing in the correct model space (*e*.*g*., with the correct number of selected alleles) gave more accurate predictions of the selection coefficients and of the positions of the sites under selection. We tested the prediction of selection coefficients in simulations where we varied the selection coefficients of the selected sites *s* from 0.005 to 0.05, and the distance in Morgans between the selected sites from 0.005 and 0.05. In most simulated scenarios, the selection coefficient was considerably overestimated if only a single selected site was fit, as the effects of linkage between selected sites were not considered. This effect was most prominent when the two simulated sites were closer to each other and when their selection was strong (Fig 4).

Our method could also distinguish between dominant selective pressure and additive selective pressure in certain admixture and selection scenarios (S4 Fig), although this was much more sensitive to the demographic parameters. We also obtained more accurate estimated selection coefficients in simulations including dominance when we model that site as having dominant, rather than additive, fitness effects (S5 Fig). For relatively recent admixture events (*t* = 100) and lower selection coefficients (*s* ≤ 0.02), this effect was most prominent, with the selection coefficients overestimated by nearly 100% when optimizing an additive model on simulations of a site under dominant selection. However, our method generally performs poorly in inferring dominance (S4 Fig), and we caution against strong interpretation of results obtained from this approach.

**Fig 5:**
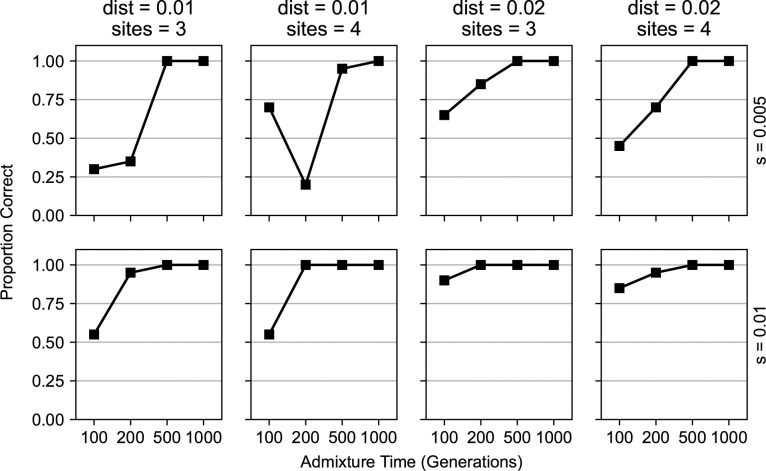
Performance of our method in detecting multiple nearby sites under selection. We evaluated the ability of AHMM-MLS to distinguish three-site and four-site models from models with one fewer site. We ran simulations of admixed populations with an admixture proportion of 0.2, and with varying selection coefficients (0.005, and 0.01 from top to bottom), distances between selected positions (0.01 Morgans for the two columns on the left, and 0.02 Morgans for the two panels on the right), number of introgressed selected sites (three for the first and third columns, four for the second and fourth columns), and time since admixture (100–1000 generations). For the 32 different scenarios, we simulated both null and alternative models, where the null model had one fewer selected site. The points on the lines indicate the proportion of alternative model simulations in which the null model was correctly rejected.

AHMM-MLS could accurately determine the number of linked sites under selection (Fig 5). In simulations, we varied the number of sites to be tested (3 to 4), the distance between the sites (1 to 2 centimorgans), the selection coefficients of the introduced sites (*s* = 0.005 to *s* = 0.01) and the number of generations since the admixture pulse (*t* = 100 to *t* = 1000). The simulated populations were the result of an introgression event where the minor population (*m* = 0.2) introduced multiple alleles spaced 1 to 2 centimorgans apart undergoing positive additive selection. For simulations where the time since the admixture pulse was around 500 generations or more, we could reliably estimate the correct number of sites. This implies that our method will be appropriate for many admixed populations including the population of sub-Saharan *D*. *melanogaster*, and the population of *P*. *italiae* that we consider below, but that caution is warranted for application to recently admixed populations with moderately weak selection.

### Effect of small population size

For relatively moderate or long times since admixture (such as our default 500 generations) and with moderate selection coefficients (*s* = 0.005–0.05), our method starts to perform poorly when the effective population size of the admixed population drops below 2000 individuals (S1 Fig). We ran forward simulations of admixed populations with various numbers of individuals, and found that the ability to distinguish between two-site and single site models was severely limited once the number of individuals dropped below 2000 (S1A Fig). The accuracy of the inferred locations and selection coefficients of the two simulated sites were also hampered when the number of individuals was below 2000 (S1B and S1C Fig). This is because our method assumes an infinite population size, with no genetic drift, and when populations are too small and admixture is relatively ancient, this assumption will produce suboptimal results. This may be an issue when studying ancient human populations, as these populations tend to have a low effective population size, although some estimates for the effective population size of the Eurasian population that admixed with Neanderthals place it right at the edge of what our program can handle (Ne = 2,100) [58]. For larger admixed populations, such as *e*.*g*., some species of *Heliconius* butterflies (Ne = 792,000) [59], we expect the population size to be large enough for the effects to be negligible.

### Likelihood approximation

To reduce the time taken to optimize each proposed model, we only calculate the effects of a selected site on the transition probabilities between two ancestry informative sites if those sites are less than a specified distance from the selected site (with a default of 2 centimorgans). When this distance is smaller than the distance between two sites, and those sites have a strong selection (*e*.*g*., selection coefficient of 0.05), then the selection coefficients for these sites can be overestimated (S6 Fig). We recommend increasing this distance when estimating models with strong selection. We also only calculate the transition probabilities between every k-th pair of adjacent ancestry informative sites, as an adjustable parameter (default of 4). We found that calculating the transition probabilities for only every k-th pair of adjacent ancestry informative sites does not affect the inference of selection coefficients when k is relatively small. If the local ancestry must be decoded for a given MLS model, then we calculate the effects of sites throughout the entire chromosome. This can be costly, as the size of the matrix ***M*** is exponential with respect to the number of sites on each tracked haplotype, so we don’t recommend doing this for models with more than 6 sites. We instead recommend that users split the model into pieces with sites that are decently separated, as we have done with chromosome 3R of *D*. *melanogaster* below.

### Robustness to demographic model misspecification

We ran simulations of two nearby selected sites and, in different simulations, we misspecified both the time since admixture and the admixture fraction by factors of 0.5, 0.8, 1.2, and 2. As the true time since admixture increased, our method was generally more robust to time misspecification. When the true time since admixture was 500 generations or higher, misspecifying by a factor of 0.5 or 2 produced only small effects for the ability of AHMM_MLS to correctly support the alternative model (S7 Fig). The estimated strength of selection was affected when the true time since admixture was small (*t* = 100), with most estimated selection coefficients being off by more than 50% (S8 Fig). This effect decreased as the true time since admixture increases to 500 or 1000, where most estimated selection coefficients had an error less than 30%. When the true admixture fraction was relatively small (m ≤ 0.2), our method was not strongly impacted by misspecifications of the admixture fraction by a factor of 0.5 or 2 (S9 Fig).

### Robustness to recombination map misspecification

Recombination occurring between linked selected sites allows us to discern their individual effects, and recombination around a selected site allows us to infer its location and strength. As such, a misspecified recombination map may confound our method’s ability to infer the number of selected positions and their selection parameters. To address these potential confounding effects, we ran simulations of two cases of recombination map misspecification.

In both of these cases we ran simulations of populations which admixed 500 generations ago, with an admixture proportion of 0.2, with a two-site or a single site selection model, and used our method to discriminate between these two scenarios. In the first case, we scaled the provided recombination rate in 100kb regions by varying amounts, to test the effect of a recombination map with high error rates. We found that our method was robust to these errors in simulations of two-site versus single site tests. Even when the error rates of the recombination maps were up to 75%, the two scenarios were accurately discriminated, and the location and selection coefficients were accurately inferred (S10 Fig). In the second case, we simulated cases where the recombination map had systematic correlated errors. We tested this by scaling the entire recombination map by a varying scalar, in simulations like those just described. We found that we could still accurately discriminate between the two cases, even with the recombination map scaled by up to 2 times the simulated rate (S11 Fig). The locations were accurately confirmed but the selection coefficients had some bias. When the map was scaled by 2x, the selection coefficients were inferred to be about 150% of their simulated values, and when the map was scaled by 0.5x, they were about 75% of their simulated values.

### Recurrent migration and selection on a cline

Our method assumes that the admixed population is the result of a single admixture pulse happening at some point in the past, and that the resulting admixed population receives no subsequent migration after that pulse. These assumptions may be difficult to meet, so we explored the effects of applying our tools to two simulated cases that break these assumptions. In both cases, we tested our method’s ability to distinguish between the presence of two selected positions and a single selected position. In the first case, we simulated ancestry on a cline, where we sample the center of the cline at various times after initial hybridization (*t* = 100, 200, 500, 1000) (S12 Fig). The clines were simulated as 20 sub-populations arranged on a line, with half of the subpopulations originating from one ancestral population and the other half from another. These sub-populations had a migration rate of 5% per generation between adjacent sub-populations. We found that our method could detect the two selected positions as well as their locations once 500 generations had passed since initial hybridization. However, even after 500 generations, the selection coefficients were still generally underestimated. In the second case, we explored the effects of recurrent migration. We simulated populations with our standard demographic parameters, 500 generations since admixture and an admixture fraction of 0.2, with varying amounts of migration from the ancestral populations into the admixed population (S13 Fig). We found that migration rates of greater than 0.0005 per generation tended to confound the signal of two-locus selection, and biased the estimation of selection coefficients.

### Effect of epistatic interactions between loci

Our method does not model epistatic interactions, which occur frequently in admixed populations. To investigate how our method reacts to epistatic interactions, we simulated simple cases of epistasis between two distant loci on a single chromosome. These populations were the result of an admixture event occurring 500 generations ago, with an admixture proportion of 0.5, and we simulated recessive epistasis with a strength of *s* = 0.05 and *s* = 0.1, and dominant epistasis with a strength of *s* = 0.01 and *s* = 0.05. We used our method to fit a model including both of these sites, and inferred their selection coefficients as well as their locations. Unsurprisingly, the selection coefficients were underestimated, as our method is treating these sites as independent selected positions (S14 Fig), but the locations were still accurate. Our method infers that one of the sites is positively selected, while the other one is negatively selected, and cannot discern the interaction between them.

## Applications

### Multi-locus selection in *Drosophila melanogaster*

In order to investigate the potential impacts of interference between linked sites on inferences of natural selection in real data, we applied our method to chromosome 3R of an admixed population of *Drosophila melanogaster* from South Africa. This population shows signals of admixture which have been noted in previous studies [44,45,60]. The admixture history is consistent with a one-pulse model, with admixture parameters that suggest that this population is suitable for our program [45]. In a previous study, we found evidence that chromosome 3R may contain multiple nearby selected alleles [43]. This study had found 13 putative sites under selection on 3R, most of which were fewer than 5 centimorgans away from another selected site. Here we fit models with multiple nearby selected positions, to determine whether and to what extent interference may have impacted our prior estimates of the number of selected sites and their selection coefficients. We used publicly available datasets of *D*. *melanogaster* collected from South Africa [61]. In a previous study, the data was prepared so that it could be analyzed by the AHMM programs [43]. This included removing the known large chromosomal inversions found on some of the chromosome arms [60,62,63]. We used a publicly available fine-scale recombination map of chromosome 3R [64]. However, we note that differences in the assumed map and the true recombination rates in this population may impact the accuracy of inferences we obtained here. We used the FlyBase sequence coordinates converter to convert assembly 5 base pair coordinates to assembly 6 [65].

### Obtaining a demographic model

We first used AHMM [44] on chromosome arm 3L of this population of *D*. *melanogaster*, to estimate a demographic model for this population. This is the other arm on chromosome 3, opposing the arm 3R, and it showed no evidence for the presence of alleles that experienced strong positive selection during admixture [43]. We inferred the admixture fraction and admixture time, obtaining values (*m* = 0.138 and *t* = 466) similar to what we obtained in prior work, which inferred these parameters on 3R directly [45], but we note that here the admixture fraction is slightly lower and the number of generations since admixture is slightly higher. Both differences are consistent with the notion that the presence of selected positions along other chromosome arms may have slightly impacted our prior demographic modeling efforts [44]. We do not account for other forms of selection that may be present, such as background selection, but the demographic inference method is generally robust to a large range of weak selection effects [44]. This supplied an estimated demographic history that we used as a baseline in our models of selection.

### Identifying candidate selected positions

**Fig 6:**
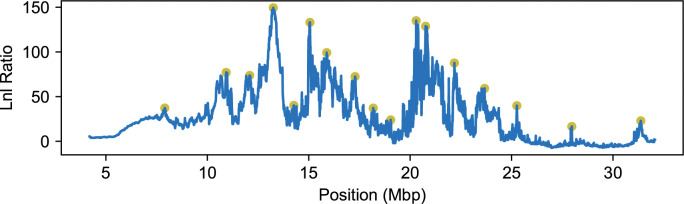
Candidate selected positions on chromosome 3R of *D*. *melanogaster*. Chromosome 3R of *D*. *melanogaster* shows signs of many nearby selected sites that are in close linkage. The likelihood ratio outputs of AHMM-S, which test each site for additive selection using a single selected site model, indicate high variation in models of natural selection (blue). Using a simple peak finding algorithm, we identified 17 sites that may be experiencing selection in this admixed population (yellow dots).

We ran AHMM-S [43] to evaluate evidence for positive selection for cosmopolitan ancestry along chromosome arm 3R (Fig 6). This program evaluates a neutral model and optimizes an additive selection model and outputs the likelihoods for each. At each position, we recorded the log likelihood ratio between each model. To identify local optima, we performed a simple peak finding algorithm, where we recorded each site that had the highest maximum likelihood of the nearest 1,400 sampled sites and a log likelihood ratio above 15. This gave us 17 candidate selected positions to examine, which we ordered by decreasing likelihood ratio. We then applied the iterative procedure described above to produce a multi-locus selection model, fixing the position of each selected position and optimizing their selection coefficients under an additive selection model. When performing simulations under the null model to determine the expected likelihood ratio distributions, we used a population size of 10,000. This is probably smaller than the effective population size in natural *D*. *melanogaster* populations. Presumably, because smaller populations are more impacted by drift, we expect that this will be a conservative choice when evaluating evidence for positive selection in this natural population (S2 Fig). If the true population size is larger in reality, additional candidate positions might exceed the significance threshold, but the selection coefficients are not expected to change substantially.

**Fig 7:**
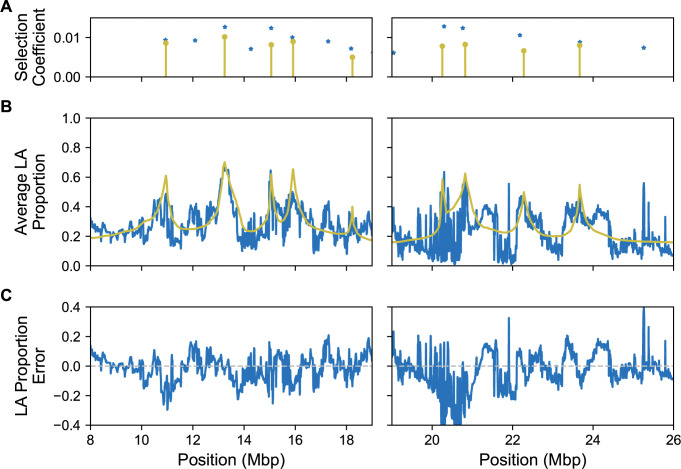
The local ancestry (LA) expected from the model roughly follows the estimated local ancestry of the samples. Due to our method having a time complexity that is exponential with respect to the number of sites, we only calculate these expected local ancestries for half of the chromosome arm at a time (subdivided on the left and right). **(A)** The identified sites and their selection coefficients from our method (yellow) along with 14 out the 17 candidate selected positions from AHMM-S (blue). **(B)** The mean local ancestries from the samples (blue) and the local ancestries expected from the model (yellow). **(C)** Error between the model’s predicted local ancestry and what we infer from the empirical data.

After applying the procedure to iteratively construct a model of multiple selected sites for chromosome arm 3R, we identified 9 selected sites (Fig 7). We then fine-tuned the position of these sites, and then re optimized the selection coefficients to arrive at our final model (Table 1). Among the 9 sites included in the multi-locus model is the Ace locus on 3R, which has common alleles that confer resistance to insecticides [66]. Our model does not include the *CHKov1* gene on 3R, which was also not recovered from our lab’s previous study [43], perhaps because the variants responsible for infection resistance are thought to have been present in both ancestral African populations [67,68]. Comparing our model to our previous approach that treated each site as a separate hypothesis test, we found that selection coefficients on 3R may have been overestimated. Indeed, the selection coefficients estimated by AHMM-S were up to 49% higher than those found by our method (Table 1). Presumably this occurs because each positively selected site is hitchhiking to an extent on linked positively selected sites, thereby increasing their frequencies in aggregate to a larger degree than would be expected given the same selection coefficient at an isolated selected position. This effect would also confound other methods to estimate the selection coefficient, such as those based on excess of local ancestry. We therefore conclude that linkage among selected positions has likely impacted analyses of selection during admixture. More generally, the tool we present in this work is a useful approach for disentangling these potentially complex effects.

**Table 1:** Comparison of selection coefficients inferred for selected sites by AHMM-MLS and AHMM-S.

Approximate base pair position of selected site	Selection Coefficient estimated by AHMM-MLS	Selection Coefficient estimated by AHMM-S	Percentage change	AHMM-S likelihood ratio	AHMM-MLS Likelihood ratio with respect to null model
7902645	Not supported	0.0073		37.22	8.17
10946217	0.0086	0.0093	8%	72.82	54.09
12088137	Not supported	0.0093		73.74	6.31
13239296	0.0102	0.0125	23%	144.50	97.56
14265104	Not supported	0.0071		40.13	0.03
15062567	0.0082	0.0122	49%	130.89	82.73
15914622	0.0090	0.0098	9%	93.24	56.21
17284518	Not supported	0.0090		72.46	23.26
18229524	0.0050	0.0056	12%	21.92	21.46
19040446	Not supported	0.0061		23.95	6.82
20255076	0.0078	0.0116	49%	89.97	107.26
20820785	0.0082	0.0122	48%	116.61	39.90
22277138	0.0066	0.0094	41%	71.90	31.61
23665629	0.0080	0.0088	10%	57.31	34.14
25258231	Not supported	0.0074		39.85	18.99
27968043	Not supported	0.0053		16.56	0.52
31371682	Not supported	0.0056		22.90	5.53

**Table 1.** Comparison of selection coefficients inferred for selected sites on 3R of South African *D*. *melanogaster* in AHMM-MLS and AHMM-S.

Additionally, we found that many positions that when analyzed individually are consistent with positive selection on admixed ancestry, are no longer supported when we consider the effects of linkage between selected sites. Of the original 17 candidate sites we identified using AHMM-S, 8 were not included in the final model selected by our iterative procedure. As might be expected, these 8 positions are primarily those with lower selection coefficients as estimated by AHMM-S (Table 1). However, when analyzed individually using AHMM-S, we found that each had a large likelihood ratio when comparing a single site selection model to a neutral model (16.56–73.74, Table 1). Support for sites that are relatively distant from other selected positions, such as the candidate site at 31371682 base pairs, were still impacted. This result emphasizes the importance of evaluating multi-locus selection models to capture the evolutionary dynamics of natural selection in admixed populations.

### Application to *Passer italiae*

We applied our method to another admixed system to confirm its wide applicability to other recombining admixed populations. This system is a population of Italian Sparrows (*Passer italiae*), which is an admixed population with *P*. *domesticus bactrianus* and *P*. *hispaniolensis* ancestry [69]. Importantly for our work, this population has a previously estimated demographic history [70], and the ancestry tract length distribution in this population shows a single peak, possibly consistent with a single pulse admixture (S15 Fig). We collected whole genome resequencing data for 122 specimens of European sparrow (*Passer domesticus bactrianus*, *P*. *italiae and P*. *hispaniolensis*) from the SRA repository [69,70]. We then used the SNParcher workflow [71] to generate a multi-sample VCF. In short, SNParcher trims the fastq files using fastp and aligns the trimmed reads to the *P*. *domesticus* genome (GCA_001700915.1) using BWA mem [72], duplicate reads are marked and removed using sambamba [73] and a multisample VCF is produced using GATK [74]. Individuals used to construct the parental panels were identified using ancestry estimates from Admixture analysis by selecting the subset of individuals displaying nearly complete, 0.95 or greater, ancestry from one of the two populations [75]. We obtained the admixed population by selecting the subset of individuals with a very similar admixture fraction (0.25–0.31) (S1 Table). This is necessary because samples in the whole dataset were taken across a geographic range and display different ancestry fractions in some cases. For admixed panels we used a flat recombination rate of 2 centimorgan per Mbp. For null model simulations, we used MaCS [55] to simulate genotypes for unadmixed individuals based on the demographic history of the parental species [70] using the following command line:

macs 400 112673505 -t 0.00409 -r 0.00772 -I 2 200 200 0 -n 2 0.951 -en 0.0182 1 0.0144 -en 0.0327 1 0.327 -en 0.0293 2 0.541 -ej 2.368 1 2 -en 2.368 2 0.0562

Similarly to the *D*. *melanogaster* population, we simulated admixed populations with an effective population size of 10,000.

Many chromosomes show evidence of multiple sites under selection for *P*. *domesticus bactrianus* ancestry according to AHMM-S, making this a suitable system for use with our method. In particular, chromosome 1 shows evidence of a few strong peaks in the likelihood ratio, potentially indicating the presence of selected sites throughout the whole chromosome, which may be in linkage with each other. Because we lacked a neutral chromosome arm on chromosome 1, we used AHMM [44] on the chromosomes 1 through 6 and 16 to infer the demographic parameters of the admixed population. We chose these chromosomes for their larger size, as smaller chromosomes may have recombination rates that differ from our assumed uniform rate and could affect our demographic inference. We obtained values for the admixture fraction and time since admixture that were suitable for our program (*m* = 0.322, and *t* = 438).

### Selection model choice for *P*. *italiae*

**Fig 8:**
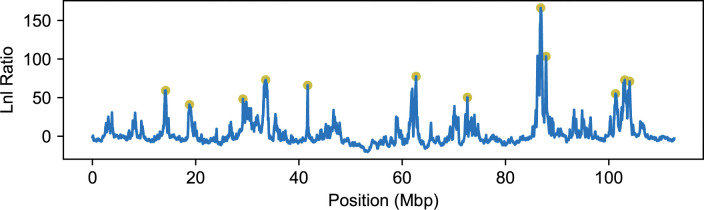
Candidate selected positions for chromosome 1 of *P*. *italiae*. The maximum likelihood of a single site model fit for every sampled position along the chromosome (blue), and the local maxima that we take to be candidate selected positions for our iterative method (yellow dots).

**Fig 9:**
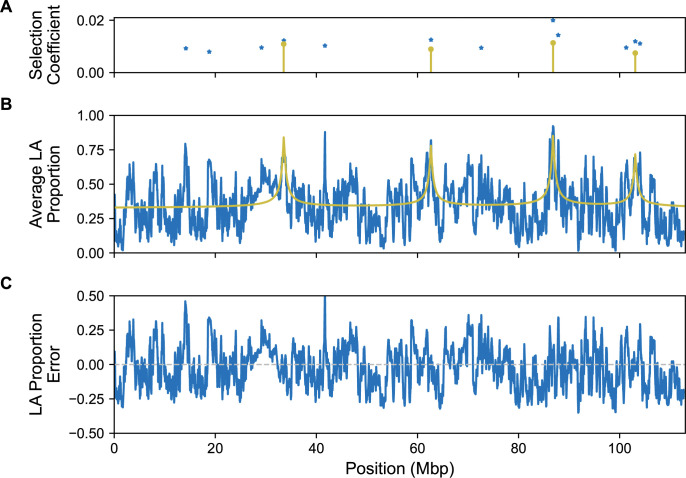
Local ancestry expected from our model follows broad peaks in the local ancestry inferred from the *P*. *italiae* population. **(A)** Selection coefficients of sites identified using AHMM-S (blue) and AHMM-MLS (yellow). **(B)** Expected local ancestry proportion from multi-locus model (yellow) and the mean local ancestry from the samples (blue). **(C)** Error between the model’s predicted local ancestry and what we infer from the empirical data.

We carried out the same procedure for choosing and evaluating selected positions as we did for *D*. *melanogaster*. Candidate selected positions on chromosome 1 were chosen using AHMM-S, where positions with the highest maximum likelihood of the nearest 300 sites and a log likelihood ratio above 40 were candidates (Fig 8). Even with a high log likelihood ratio cutoff, most candidate positions were rejected by our iterative method. Of the 12 candidate positions, only 4 were identified in our multi-locus model (Fig 9). As was the case with the *D*. *melanogaster* population, the selection coefficients of the candidate positions appear to have been overestimated when fitting single site models using AHMM-S, when compared to the multi-site model that account for linkage using AHMM-MLS (Table 2).

**Table 2:** Multi-locus model inferred for chromosome 1 of *P*. *italiae*.

Approximate base pair position of selected site	Selection Coefficient estimated by AHMM-MLS	Selection Coefficient estimated by AHMM-S	Percentage change	AHMM-S likelihood ratio	AHMM-MLS Likelihood ratio with respect to null model
14180647	Not supported	0.0093		59.15	28.15
18805566	Not supported	0.0080		40.89	18.57
29151015	Not supported	0.0095		48.19	19.63
33530452	0.0109	0.0122	12%	72.99	30.08
41695798	Not supported	0.0103		65.87	18.04
62650766	0.0090	0.0125	40%	77.39	39.83
72625209	Not supported	0.0095		50.29	25.33
86810201	0.0114	0.0200	76%	166.18	80.78
87850243	Not supported	0.0143		103.37	5.61
101300767	Not supported	0.0095		55.00	10.02
103066002	0.0075	0.0119	60%	72.80	40.68
104031022	Not supported	0.0111		71.13	4.21

**Table 2.** Comparison of selection coefficients inferred for selected sites on chromosome 1 of *P*. *italiae* with AHMM-MLS and AHMM-S.

### Caveats

Although our method provides a promising means for quantifying and investigating the impacts of interference and natural selection in admixed populations, there are several important caveats. First, the state space of multi-locus selection models is extraordinarily complex, and there is no evaluation procedure that could exhaustively attempt all possibilities. For example, even in the scenario that we considered for *D*. *melanogaster* with 17 candidate selected positions, there are 2^17^ (~130,000) possible sets of sites in multi-locus selection models. This is an intractable number of models to test, as the time taken to optimize a single model with three sites can take 10 minutes on a single core with an M2 processor. The iterative procedure we present is an appealing way to prioritize model space and we expect that it will perform well in a variety of scenarios, but undoubtedly there are other plausible models that we could not evaluate. Second, our approach will accommodate scenarios where there are a modest set of loci of relatively large effect. However, some authors have proposed that the aggregate effect of hundreds of weakly selected linked mutations might shape the landscape of admixed ancestry in natural populations [9]. Our approach is not well suited to such scenarios because each site is unlikely to reach significance in itself and because the time taken to compute the expected transitions for each model is exponential with respect to the number of sites. Third, our approach is also not suitable for populations with a small number of individuals (Ne < 2000), or which were admixed recently (t ⪅ 200), except possibly if selection is very strong (*e*.*g*., s > 0.025). We expect that AHMM-MLS will typically perform best with populations that are only somewhat genetically divergent and where strong selection affects the dynamics of introduced alleles.

### Conclusion

Admixture has the potential to simultaneously introduce multiple linked selected sites, but this phenomenon is rarely addressed in empirical investigations. To meet this need, we created AHMM-MLS. In validating our method over simulated data, we found that it could identify multiple nearby selected sites, and estimate the selection coefficients better when the linkage between these sites was accounted for. We found that our previous study of adaptive introgression on chromosome arm 3R of an admixed *D*. *melanogaster* population may have overestimated the number and strength of selected sites along the chromosome. We similarly found that failing to account for linkage may overestimate the number and strength of selected sites in an admixed *P*. *italiae* population. Because divergent populations may introduce many selected alleles at once, analyzing the effects of linkage between these sites is critical for understanding the evolutionary dynamics of admixed populations. We hope that our method can be applied to the many examples of adaptive introgression that have already been identified, and can better quantify cases where multiple advantageous sites have been introduced at once.

## Supporting information

S1 Fig Evaluating the effects of population size. We simulated populations with admixture parameters *m* = 0.2 and *t* = 500, in which the introgressing population brought in two selected alleles with selection coefficients of 0.01. We also simulated null model cases in which only a single selected allele with the same selection coefficient was introgressed. For each of these population models, we simulated populations with varying numbers of individuals, (Ne = 100000, Ne = 10000, 5000, 2000, 1000, and 500 from left to right). **(A)** The proportion of two site simulations in which the number of sites was correctly estimated. **(B)** The position in Morgans of the two inferred selected sites (blue) and the simulated positions (black lines). The x-axis is the position of the first selected position, and the y-axis is the position of the second selected position. Each blue dot corresponds to fitting two sites on a single simulation. Both axes have been translated so that the simulated position is at 0 Morgans. **(C)** The inferred selection coefficients of the two sites (dark and light blue), and the simulated selection coefficients (black).
(EPS)

S2 Fig Finite populations skew the likelihood ratio distributions. We simulated 50 neutral admixed populations with admixture parameters *m =* 0.2, *t* = 500, and varying population sizes. On each simulation we fit a neutral model and we fit the selection coefficient of a single selected site with the location and dominance coefficient fixed. The swarm plots show the log likelihood ratio between these two models, as well as the theoretically expected chi-squared distribution when fitting a single parameter. Our model assumes an infinite population, so as the simulated population grows larger, the log likelihood ratio distribution more closely matches the theoretically expected distribution. We note that the finite population size does not fully account for the disconnect between the simulated distributions and the theoretical distribution.
(EPS)

S3 Fig Accuracy of the estimated positions of two selected sites. Each simulated chromosome was the result of an admixture event where *m* = 0.2, and the minor population introduced two nearby sites under positive additive selection with a selection coefficient of 0.01. For each of the 16 graphs, we ran 20 simulations which share the same distance between selected sites (0.005, 0.01, 0.02 and 0.05 Morgans, from top to bottom) and time since the admixture pulse (100, 200, 500, and 1000 generations). On the x-axis and y-axis are the locations of the selected sites on the chromosome in Morgans. The black lines going through the graphs are the true locations of the simulated sites.
(EPS)

S4 Fig For strongly selected positions, AHMM_MLS can distinguish between selected sites under dominant vs additive selection. Much like the simulations with two sites, we simulated 64 different introgression and selection scenarios, in which the introgressing population contributed a positively selected allele. We varied the minor ancestry fractions (0.05, 0.1, 0.2, and 0.5 from left to right), times since admixture (100–1000 generations), and selection coefficients (0.005, 0.01, 0.02 and 0.05, from top to bottom). For each introgression scenario, we ran null model simulations where the site had additive selection, and alternative model simulations where the site had dominant selection. The points on the line show the proportion of alternative model simulations in which the null model was correctly rejected.
(EPS)

S5 Fig Fitting a dominant selection model on simulations of dominant selection gives more accurate inferred selection coefficients. We inferred the selection coefficients of selected sites for the same simulations from S4 Fig. The selection coefficients inferred when fitting a dominant model (blue, *h* = 1) are much closer to the simulated value (black line) than those coefficients inferred when fitting an additive model (yellow, *h =* 0.5).
(EPS)

S6 Fig Speed ups employed in our method can affect the inference of strong selected sites that are far away. We simulated an admixed population with two sites under selection, 0.05 Morgans apart, in which both had a selection coefficient of either 0.01 (top panels), or 0.05 (bottom panels). On these simulations we fit a two site model with different speed up parameters in place. In light and dark blue are the inferred selection coefficients, and the black lines indicate the simulated selection coefficients. We altered the number of pairs of adjacent sampled sites that we skipped in the transition rate calculation, *k*, and found that it had very little effect. We also altered the radius around each site in the model where we account for the effect of that site (R = 0.1 Morgans, on the left panels, and R = 0.02, on the right panels) and found that for large selection coefficients a radius large enough to encompass both sites is required for accurate inference.
(EPS)

S7 Fig Evaluating the effects of misspecifying the time since admixture when comparing two site and single site models. We misspecified the time since admixture by a certain factor from the true simulated time when analyzing simulations with a single site under additive selection or two sites under additive selection. For the simulations with two selected sites, they were placed one centimorgan apart. In every simulation, the sites had a selection coefficient of 0.01, and an admixture proportion of *m* = 0.2. We varied the time since admixture from 100 to 1000 generations since admixture (left to right), and misspecified this time in our AHMM_MLS models by a factor of 0.5 to 2. The points on the lines indicate the proportion of two site simulations in which the single site null model was correctly rejected.
(EPS)

S8 Fig Comparing the inferred selection coefficients when misspecifying the time since admixture. We compared the inferred selection coefficients versus the simulated selection coefficients for the two site simulations from S7 Fig. In blue we show the inferred selection coefficients for one of the two sites, and in yellow we show the other. The black line indicates the simulated selection coefficients of both sites.
(EPS)

S9 Fig Evaluating the effects of misspecifying the admixture fraction when comparing two site and single site models. We misspecified the admixture fraction by a certain factor from the true simulated fraction when analyzing simulations with a single site under additive selection or two sites under additive selection. For the simulations with two selected sites, they were placed one centimorgan apart. In every simulation, the sites had a selection coefficient of 0.01, and the time since admixture was 400 generations. We varied the admixture fraction from 0.05 to 0.4, and misspecified this fraction in our AHMM_MLS models by a factor of 0.5 to 2. The points on the lines indicate the proportion of two site simulations in which the single site null model was correctly rejected.
(EPS)

S10 Fig Effects of uncorrelated recombination map errors. We simulated populations with the same demographic and selection parameters as those in the population size effect simulations. We provided a misspecified recombination map to our method in which each 100kb region was scaled by a random scalar from the range found above each column. **(A)** The proportion of two site simulations in which the number of sites was correctly estimated. **(B)** The position in Morgans of the two inferred selected sites (blue) and the simulated positions (black lines). The x-axis is the position of the first selected position, and the y-axis is the position of the second selected position. Each blue dot corresponds to fitting two sites on a single simulation. Both axes have been translated so that the simulated position is at 0 Morgans. **(C)** The inferred selection coefficients of the two sites (dark and light blue), and the simulated selection coefficients (black).
(EPS)

S11 Fig Effects of correlated recombination map errors. We simulated populations with the same demographic and selection parameters as those in the population size effect simulations. We provided a misspecified recombination map to our method which was scaled by the scalar found above each column. **(A)** The proportion of two site simulations in which the number of sites was correctly estimated. **(B)** The position in Morgans of the two inferred selected sites (blue) and the simulated positions (black lines). The x-axis is the position of the first selected position, and the y-axis is the position of the second selected position. Each blue dot corresponds to fitting two sites on a single simulation. Both axes have been translated so that the simulated position is at 0 Morgans. **(C)** The inferred selection coefficients of the two sites (dark and light blue), and the simulated selection coefficients (black).
(EPS)

S12 Fig Estimating parameters of selection on clinal hybrids. We simulated two-site selection acting on hybrids in a cline, and used our method to distinguish between two-site and single site selection. In each column, we vary the number of generations since initial hybridization after which we sample the population. These values are found at the top of each column. **(A)** The proportion of two-site simulations in which the number of sites was correctly estimated. **(B)** The position in Morgans of the two inferred selected sites (blue) and the simulated positions (black lines). The x-axis is the position of the first selected position, and the y-axis is the position of the second selected position. Each blue dot corresponds to fitting two sites on a single simulation. Both axes have been translated so that the simulated position is at 0 Morgans. **(C)** The inferred selection coefficients of the two sites (dark and light blue), and the simulated selection coefficients (black).
(EPS)

S13 Fig Effects of recurrent migration. We simulated two-locus selection in populations which received recurrent migration from the ancestral populations at varying rates per generation (top of each column). Each population had an admixture fraction of 0.2 and was sampled 500 generations after admixture. **(A)** The proportion of two site simulations in which the number of sites was correctly estimated. **(B)** The position in Morgans of the two inferred selected sites (blue) and the simulated positions (black lines). The x-axis is the position of the first selected position, and the y-axis is the position of the second selected position. Each blue dot corresponds to fitting two sites on a single simulation. Both axes have been translated so that the simulated position is at 0 Morgans. **(C)** The inferred selection coefficients of the two sites (dark and light blue), and the simulated selection coefficients (black).
(EPS)

S14 Fig Inference on epistatic loci. We simulated populations with two loci with a dominant or recessive epistatic interaction with varying selection coefficients (top of each column). Each population had an admixture fraction of 0.5 and was sampled 500 generations after admixture. **(A)** The position in Morgans of the two inferred selected sites (blue) and the simulated positions (black lines). The x-axis is the position of the first selected position, and the y-axis is the position of the second selected position. Each blue dot corresponds to fitting two sites on a single simulation. Both axes have been translated so that the simulated position is at 0 Morgans. **(B)** The inferred selection coefficients of the two sites (dark and light blue).
(EPS)

S15 Fig Distribution of ancestral tract lengths in *P*. *italiae*. Probability density estimation of lengths of tracts of contiguous ancestry in *P*. *italiae* samples from chromosomes 1, 2, 3, 4, 5, 6, and 16. The tract lengths were inferred from the Viterbi decoding of a neutral model using AHMM.
(EPS)

S1 Table *Passer* samples and their original projects. (DOCX)
