# Supplementary material for: Inferring multi-locus selection in admixed populations
Source: PLoS Genet. 2023 Nov 28;19(11):e1011062. doi: 10.1371/journal.pgen.1011062 (PMC10707604; doi:10.1371/journal.pgen.1011062)
Supplement: S1 Table — (DOCX) [file pgen.1011062.s016.docx]

| **Sample** | **Project** | **Species** | **Use** |
| --- | --- | --- | --- |
| SAMEA4785093 | PRJEB27649 | *P. domesticus bactrianus* | Parental population 1 |
| SAMEA4785094 | PRJEB27649 | *P. domesticus bactrianus* | Parental population 1 |
| SAMEA4785095 | PRJEB27649 | *P. domesticus bactrianus* | Parental population 1 |
| SAMEA4785096 | PRJEB27649 | *P. domesticus bactrianus* | Parental population 1 |
| SAMEA4785097 | PRJEB27649 | *P. domesticus bactrianus* | Parental population 1 |
| SAMEA4785098 | PRJEB27649 | *P. domesticus bactrianus* | Parental population 1 |
| SAMEA4785099 | PRJEB27649 | *P. domesticus bactrianus* | Parental population 1 |
| SAMEA4785100 | PRJEB27649 | *P. domesticus bactrianus* | Parental population 1 |
| SAMEA4785101 | PRJEB27649 | *P. domesticus bactrianus* | Parental population 1 |
| SAMEA4785102 | PRJEB27649 | *P. domesticus bactrianus* | Parental population 1 |
| SAMEA4785112 | PRJEB27649 | *P. domesticus bactrianus* | Parental population 1 |
| SAMEA4785113 | PRJEB27649 | *P. domesticus bactrianus* | Parental population 1 |
| SAMEA4785114 | PRJEB27649 | *P. domesticus bactrianus* | Parental population 1 |
| SAMEA4785115 | PRJEB27649 | *P. domesticus bactrianus* | Parental population 1 |
| SAMEA4785116 | PRJEB27649 | *P. domesticus bactrianus* | Parental population 1 |
| SAMEA4785117 | PRJEB27649 | *P. domesticus bactrianus* | Parental population 1 |
| SAMEA4785118 | PRJEB27649 | *P. domesticus bactrianus* | Parental population 1 |
| SAMEA4785119 | PRJEB27649 | *P. domesticus bactrianus* | Parental population 1 |
| SAMEA4785120 | PRJEB27649 | *P. domesticus bactrianus* | Parental population 1 |
| SAMEA4785058 | PRJEB27649 | *P. hispaniolensis* | Parental population 2 |
| SAMEA4785059 | PRJEB27649 | *P. hispaniolensis* | Parental population 2 |
| SAMEA4785061 | PRJEB27649 | *P. hispaniolensis* | Parental population 2 |
| SAMEA4785063 | PRJEB27649 | *P. hispaniolensis* | Parental population 2 |
| SAMEA4785064 | PRJEB27649 | *P. hispaniolensis* | Parental population 2 |
| SAMEA4785065 | PRJEB27649 | *P. hispaniolensis* | Parental population 2 |
| SAMEA4785066 | PRJEB27649 | *P. hispaniolensis* | Parental population 2 |
| SAMEA4785067 | PRJEB27649 | *P. hispaniolensis* | Parental population 2 |
| SAMEA4785068 | PRJEB27649 | *P. hispaniolensis* | Parental population 2 |
| SAMEA4785069 | PRJEB27649 | *P. hispaniolensis* | Parental population 2 |
| SAMEA4785090 | PRJEB27649 | *P. hispaniolensis* | Parental population 2 |
| SAMEA4785091 | PRJEB27649 | *P. hispaniolensis* | Parental population 2 |
| SAMEA4785092 | PRJEB27649 | *P. hispaniolensis* | Parental population 2 |
| SAMEA4785103 | PRJEB27649 | *P. hispaniolensis* | Parental population 2 |
| SAMEA4785104 | PRJEB27649 | *P. hispaniolensis* | Parental population 2 |
| SAMEA4785105 | PRJEB27649 | *P. hispaniolensis* | Parental population 2 |
| SAMEA4785106 | PRJEB27649 | *P. hispaniolensis* | Parental population 2 |
| SAMEA4785107 | PRJEB27649 | *P. hispaniolensis* | Parental population 2 |
| SAMEA4785108 | PRJEB27649 | *P. hispaniolensis* | Parental population 2 |
| SAMEA4785109 | PRJEB27649 | *P. hispaniolensis* | Parental population 2 |
| SAMEA4785110 | PRJEB27649 | *P. hispaniolensis* | Parental population 2 |
| SAMEA4785131 | PRJEB27649 | *P. hispaniolensis* | Parental population 2 |
| SAMEA4785132 | PRJEB27649 | *P. hispaniolensis* | Parental population 2 |
| SAMEA4785133 | PRJEB27649 | *P. hispaniolensis* | Parental population 2 |
| SAMEA4785134 | PRJEB27649 | *P. hispaniolensis* | Parental population 2 |
| SAMEA4785135 | PRJEB27649 | *P. hispaniolensis* | Parental population 2 |
| SAMEA4785136 | PRJEB27649 | *P. hispaniolensis* | Parental population 2 |
| SAMEA4785137 | PRJEB27649 | *P. hispaniolensis* | Parental population 2 |
| SAMEA4785138 | PRJEB27649 | *P. hispaniolensis* | Parental population 2 |
| SAMEA4785139 | PRJEB27649 | *P. hispaniolensis* | Parental population 2 |
| SAMEA4785140 | PRJEB27649 | *P. hispaniolensis* | Parental population 2 |
| SAMEA104363402 | PRJEB22939 | *P. italiae* | Admixed population |
| SAMEA104363403 | PRJEB22939 | *P. italiae* | Admixed population |
| SAMEA104363404 | PRJEB22939 | *P. italiae* | Admixed population |
| SAMEA104363405 | PRJEB22939 | *P. italiae* | Admixed population |
| SAMEA104363406 | PRJEB22939 | *P. italiae* | Admixed population |
| SAMEA104363408 | PRJEB22939 | *P. italiae* | Admixed population |
| SAMEA104363409 | PRJEB22939 | *P. italiae* | Admixed population |
| SAMEA104363410 | PRJEB22939 | *P. italiae* | Admixed population |
| SAMEA104363411 | PRJEB22939 | *P. italiae* | Admixed population |
| SAMEA104363412 | PRJEB22939 | *P. italiae* | Admixed population |
| SAMEA104363413 | PRJEB22939 | *P. italiae* | Admixed population |
| SAMEA104363414 | PRJEB22939 | *P. italiae* | Admixed population |
| SAMEA104363415 | PRJEB22939 | *P. italiae* | Admixed population |
| SAMEA104363416 | PRJEB22939 | *P. italiae* | Admixed population |
| SAMEA104363417 | PRJEB22939 | *P. italiae* | Admixed population |
| SAMEA104363418 | PRJEB22939 | *P. italiae* | Admixed population |
| SAMEA104363419 | PRJEB22939 | *P. italiae* | Admixed population |
| SAMEA104363420 | PRJEB22939 | *P. italiae* | Admixed population |
| SAMEA104363421 | PRJEB22939 | *P. italiae* | Admixed population |
| SAMEA104363422 | PRJEB22939 | *P. italiae* | Admixed population |
| SAMEA104363407 | PRJEB22939 | *P. italiae* | Admixed population |
| SAMEA4785070 | PRJEB27649 | *P. italiae* | Admixed population |
| SAMEA4785071 | PRJEB27649 | *P. italiae* | Admixed population |
| SAMEA4785072 | PRJEB27649 | *P. italiae* | Admixed population |
| SAMEA4785073 | PRJEB27649 | *P. italiae* | Admixed population |
| SAMEA4785074 | PRJEB27649 | *P. italiae* | Admixed population |
| SAMEA4785075 | PRJEB27649 | *P. italiae* | Admixed population |
| SAMEA4785076 | PRJEB27649 | *P. italiae* | Admixed population |
| SAMEA4785077 | PRJEB27649 | *P. italiae* | Admixed population |
| SAMEA4785078 | PRJEB27649 | *P. italiae* | Admixed population |
| SAMEA4785079 | PRJEB27649 | *P. italiae* | Admixed population |
