# Supplementary material for: Inferring multi-locus selection in admixed populations
Source: PLoS Genet. 2023 Nov 28;19(11):e1011062. doi: 10.1371/journal.pgen.1011062 (PMC10707604; doi:10.1371/journal.pgen.1011062)
Supplement: Attachment — Submitted filename: Revision Response.pdf [file pgen.1011062.s017.pdf]

We thank the editor and reviewers for thoughtful and helpful comments on our manuscript. We have thoroughly addressed all of the major comments in our resubmission. Please find below the comments reproduced in full with our responses interspersed in bold.

Dear Dr Ayala,

Thank you very much for submitting your Research Article entitled 'Inferring multi-locus selection in admixed populations' to PLOS Genetics.

The manuscript was fully evaluated at the editorial level and by independent peer reviewers. The reviewers appreciated the attention to an important problem, but raised some substantial concerns about the current manuscript. Based on the reviews, we will not be able to accept this version of the manuscript, but we would be willing to review a much-revised version. We cannot, of course, promise publication at that time.

Should you decide to revise the manuscript for further consideration here, your revisions should address the specific points made by each reviewer. We will also require a detailed list of your responses to the review comments and a description of the changes you have made in the manuscript.

If you decide to revise the manuscript for further consideration at PLOS Genetics, please aim to resubmit within the next 60 days, unless it will take extra time to address the concerns of the reviewers, in which case we would appreciate an expected resubmission date by email to plosgenetics@plos.org.

If present, accompanying reviewer attachments are included with this email; please notify the journal office if any appear to be missing. They will also be available for download from the link below. You can use this link to log into the system when you are ready to submit a revised version, having first consulted our Submission Checklist.

To enhance the reproducibility of your results, we recommend that you deposit your laboratory protocols in protocols.io, where a protocol can be assigned its own identifier (DOI) such that it can be cited independently in the future. Additionally, PLOS ONE offers an option to publish peer-reviewed clinical study protocols. Read more information on sharing protocols at https://plos.org/protocols?utm_medium=editorial-email&utm_source=authorletters&utm_campaign=protocols

Please be aware that our data availability policy requires that all numerical data underlying graphs or summary statistics are included with the submission, and you will need to provide this upon resubmission if not already present. In addition, we do not permit the inclusion of phrases such as "data not shown" or "unpublished results" in manuscripts. All points should be backed up by data provided with the submission.

While revising your submission, please upload your figure files to the Preflight Analysis and Conversion Engine (PACE) digital diagnostic tool. PACE helps ensure that figures meet PLOS requirements. To use PACE, you must first register as a user. Then, login and navigate to the UPLOAD tab, where you will find detailed instructions on how to use the tool. If you encounter any issues or have any questions when using PACE, please email us at figures@plos.org.

PLOS has incorporated Similarity Check, powered by iThenticate, into its journal-wide submission system in order to screen submitted content for originality before publication. Each PLOS journal undertakes screening on a proportion of submitted articles. You will be contacted if needed following the screening process.

To resubmit, use the link below and 'Revise Submission' in the 'Submissions Needing Revision' folder.

We are sorry that we cannot be more positive about your manuscript at this stage. Please do not hesitate to contact us if you have any concerns or questions.

Yours sincerely,

Nicolas Bierne
Academic Editor
PLOS Genetics

Xiaofeng Zhu
Section Editor
PLOS Genetics

Dear Dr Ayala:
We have received three thoughtful reviews of your manuscript entitled "Inferring multi-locus selection in admixed populations". All three reviewers and I enjoyed the manuscript and agreed that, despite the limitations and caveats of the method, this is a valuable step towards better analysing adaptive introgression in admixed populations.
The reviewers did make a number of recommendations to investigate some of the confounding effects in more detail and to improve the clarity of the manuscript. Most importantly, they ask you to better explore and discuss the effects of recombination and demography (including when they are misinfered) and the neglect of epistasis. Although you have done a good job of evaluating the performance of the method, some questions remain about its applicability to real data in a general context, e.g. what if the recombination map or demography is incorrect or biased (reviewers 1 and 3)? In admixed populations, there is often contrasting selection between different genomic regions, with genome-wide purging of the minor parent ancestry when ancestry variance is high in the first few generations after the admixture pulse. Negative and epistatic selection can make the admixture rate (m) different between genomic regions (with different recombination rates or density of selected genes), and this can be a problem unless the method is robust to deviations from the true local m on the chromosomal stretch under study. Adaptive introgression can sometimes be seen more as a resistance to this purging erosion, and this can produce the multi-peak pattern you are interested in (referee 2).
Finally, you should include some more details directly in the manuscript that we need to find in your previous work. Ancestry_HMM-S has been published recently and you should be more explicit about the difference between the two methods (not just that AHMM-S is mono-locus and AHMM-MLS is multi-locus): your numerical method implies inferring t and m from genomic regions assumed to be neutral (and this will need to be better adressed in the revised version), whereas, as far as I understand, the Shchur et al. approximations allow all-in-1 inference in AHMM-S. Could you also clarify what you mean by ancestry informative positions? The Drosophila example should also be better clarified. We want to know what the other chromosomes look like, and we need to read previous manuscripts to get that information. The data should be accessible and perhaps some supporting figures should be provided to describe the system, e.g. population differentiation, linkage disequilibrium blocks, Marey maps, etc. In Svedberg et al. selection for resistance was discussed at length, whereas it is missing here (reviewer 3). Still, it remains puzzling why resistance genes are clustered on chromosome 3R (and each have s~0.01 with admixture proportions ~0.6) while some other chromosome arms are free from selection for resistance genes. In the end, it looks like you developed AHMM-MLS to improve the inference made on this system with AHMM-S, and this does not show that your method is of interest in other systems where a few adaptive introgression breakthroughs are observed, or where positive, negative and epistatic selection is all over the genome. It would be more impactful to apply it to another system.
Please try to address all of the referees' concerns in a revised version. I'm looking forward to reading it.
Best regards,
Nicolas Bierne

We thank the editor for their assessment. We agree that applying our method to another system would make the manuscript more impactful, so we have included an analysis on an admixed population of *Passer italiae*. The *Drosophila* system that we examine has been explored in other works, (Pool et al. 2012, Lack et al. 2015, Corbett-Detig and Nielsen 2017, Medina et al. 2018, Svedberg et al. 2021), and we believe that including significantly more details on this system would distract from the main points of this work. Other points brought up by the reviewers have been addressed below.

Reviewer's Responses to Questions

Comments to the Authors:
Please note here if the review is uploaded as an attachment.

Reviewer #1: The manuscript by Ayala and Corbett-Detig presents a HMM method to infer the strength of location acting on admixed ancestry that can be applicable to genome-wide data. One important advance in their method is to model natural selection at multiple sites simultaneously. Through simulations, they show that if in truth multiple sites are experiencing selection, but a model with only one site under selection is fit to the data, the strength of selection will be overestimated. Thus, modeling multiple sites under selection is important for accurate inference.

Overall, I really enjoyed this manuscript. I think the method is quite clever and innovative. It also addresses a significant research question—how to best infer selection on alleles recently brought into a new population due to admixture. The authors have done a nice job evaluating the performance of their method under different circumstances using simulations and show that it performs well. Applying their method to Drosophila data, the authors found 9 selected sites on chromosome arm 3R and that the selection coefficients in previous work were likely overestimated due to the cryptic presence of multiple linked SNPs experiencing selection.

While I think the authors did a nice job on testing robustness to violations of the model and mis-specifying certain parameters, one area where I think more work is needed relates to the recombination rate in the region of interest. In particular, based on my understanding, it seems that both selection and recombination affect and change haplotype frequencies in the model. As such, accurate estimates of the recombination rate must be needed. This raises several questions that the authors should try to address. First, how do the authors recommend that uncertainty in the recombination rate be dealt with when using their method? Second, I think the authors should undertake additional simulations where the true recombination rate used to generate the data is different from the rate assumed in the inference. How much does performance change in this situation? In particular, is the false-positive rate elevated when the wrong recombination rate is assumed in the inference? Is the location of the selected site(s) mis-inferred in this situation? I think more exploration of recombination is necessary.

To address the potential confounding factors that a misspecified recombination map might produce, we ran two additional tests where we supplied our program with an inaccurate map. In the first test, we scaled the recombination rate in 100kb regions by a varying error, to test the likely case of a recombination map with uncorrelated errors. We found that our method was robust to these errors in simulations of two site versus one site tests. Even when the error rates of the recombination maps were up to 75%, the two cases (single vs two site) were accurately discriminated, and the location and selection coefficients were accurately inferred. Below is a new supplementary figure we have added to the manuscript.

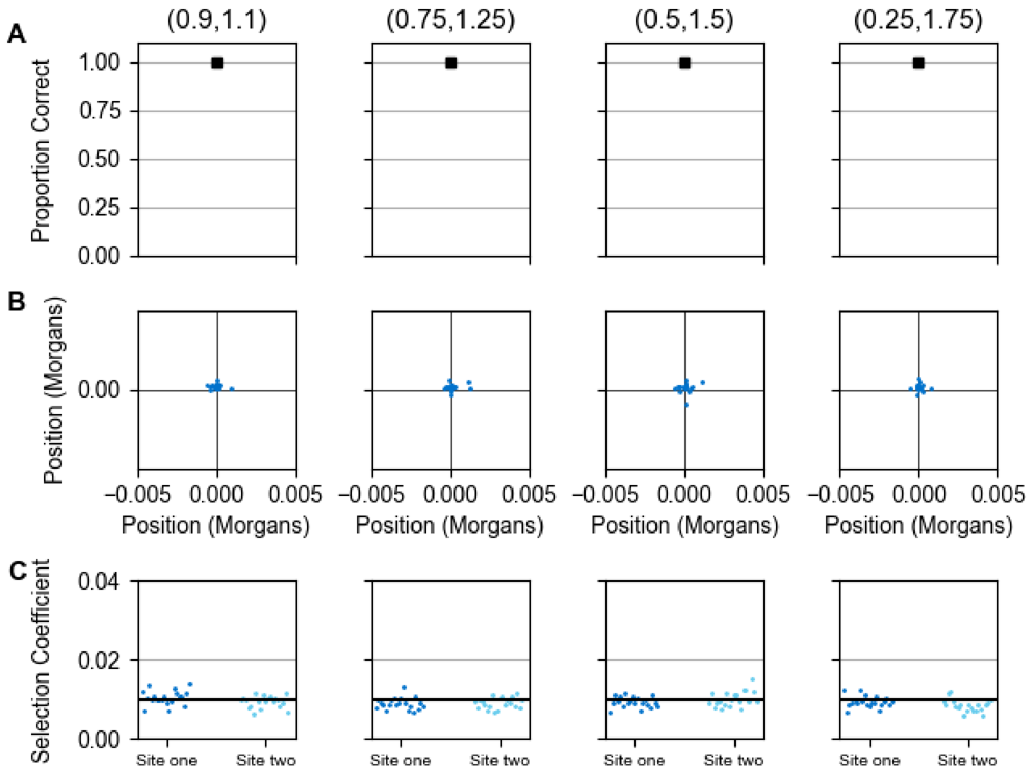

S11 Fig. Effects of uncorrelated recombination map errors. We simulated populations with the same demographic and selection parameters as those in the population size effect simulations. We provided a misspecified recombination map to our method in which each 100kb region was scaled by a random scalar from the range found above each column. (A) The proportion of two site simulations in which the number of sites was correctly estimated. (B) The position in Morgans of the two inferred selected sites (blue) and the simulated positions (black lines). The x-axis is the position of the first selected position, and the y-axis is the position of the second selected position. Each blue dot corresponds to fitting two sites on a single simulation. Both axes have been translated so that the simulated position is at 0 Morgans. (C) The inferred selection coefficients of the two sites (dark and light blue), and the simulated selection coefficients (black).

We also simulated cases where the recombination map had systematic correlated errors. This might occur, *e.g.,* if a panel used to measure a recombination map included individuals with heterozygous inversions. We tested this by scaling the entire recombination map by a varying scalar, in simulations similar to those just described. We found that we could still accurately discriminate between the two cases, even with the recombination map scaled by up to 2 times the simulated rate. The locations were accurately confirmed but the selection coefficients had some bias. When the map was scaled by 2x, the selection coefficients were inferred to be about 150% of their simulated values, and when the map was scaled by 0.5x, they were about 75% of their simulated values.

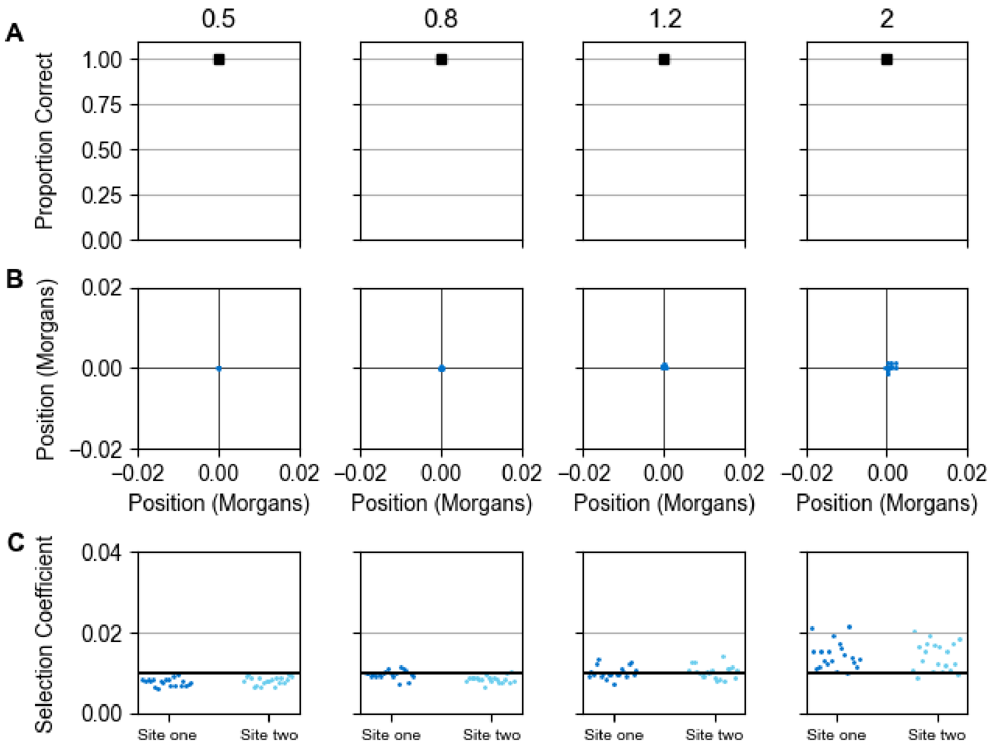

S12 Fig. Effects of correlated recombination map errors. We simulated populations with the same demographic and selection parameters as those in the population size effect simulations. We provided a misspecified recombination map to our method which was scaled by the scalar found above each column. (A) The proportion of two site simulations in which the number of sites was correctly estimated. (B) The position in Morgans of the two inferred selected sites (blue) and the simulated positions (black lines). The x-axis is the position of the first selected position, and the y-axis is the position of the second selected position. Each blue dot corresponds to fitting two sites on a single simulation. Both axes have been translated so that the simulated position is at 0 Morgans. (C) The inferred selection coefficients of the two sites (dark and light blue), and the simulated selection coefficients (black).

We've added these analyses to the manuscript starting on line 461.

I also have a few more minor comments:

1) Line 145-146: "Forward equations" are mentioned. I assume that the authors are referring to the forward algorithm to calculate the probability of the data under the model for HMMs. Maybe specify this and provide a reference here for the uninitiated reader.

We've made the change and added the reference.

2) I really like Figure 1!

Thanks!

3) Methods, haplotype frequency matrix: I think a cartoon figure of the H matrix might make the presentation a bit clearer.

We've placed a cartoon figure (now Figure 1) in the manuscript to explain the H, D and M matrices and how they relate. We agree that this improved the clarity of our presentation.

4) Methods, While the authors do a nice job explaining different parts and components of their method, I think some additional explanation about how the method would be applied to genome-wide data would be beneficial. For example, is the same M matrix used for all sites across the genome? Or, are different Ms computed? How does this vary with recombination rate? Is M recomputed for each s and h value?

For each of these cases M would have to be recomputed. M depends on the position of both the sample sites and the selected positions, as well as their selection coefficients. Despite this, a model of specific selection coefficients can be computed on around 30,000 sampled sites in a matter of seconds, making this method ideal for genome-wide data. We have added a sentence in the Method section explicitly stating this consideration (line 224).

Reviewer #2: In this manuscript, Ayala & Corbett-Detig present a new approach—an extention of the PI's previous work—to detect multi-locus selection along chromosomes. The main advance here is that the approach accounts for linkage between selected sites to identify them an estimate their selection coefficients. The approach has many limitations and caveats that are duly acknowledged by the authors, for example not accounting for epistasis (ubiquitous in hybrids), assuming a single admixture pulse, and performs poorly in recent hybrid populations (few gens of admixture), at estimating dominance, and with small-effect alleles. However, the authors also show clearly that existing approaches are likely to greatly overestimate selection. This approach is clearly a step forward for the field.

The only major comment that I have is that I'd like to see a bit more investigation into epistasis. As noted above, the major limiting assumption of the work is that it does not consider epistasis, which is likely rampant in admixed populations. However, this is a common assumption in some key theoretical models about selection in admixed populations (e.g., Veller et al. Evolution 2023) so should by no means preclude publication. However, as the goal is for this approach to be applied to real datasets, I would like to see a brief foray into some very simple epistasis in the validation stage. If there are recessive DMIs, for example, what do the outputs of the model look like?

This is a great point. To explore how our method reacts to epistatic selection, We ran simulations of two different epistatic interactions with varying selection coefficients. We simulated both dominant and recessive epistasis, and found that our method was able to detect selection at the interacting sites. Our method is not able to discern epistatic interactions from non-epistatic selection as it cannot explicitly model epistatic interaction. The locations of the sites were accurately inferred, but the selection coefficients were underestimated relative to the strength of selection that would be observed in an individual homozygous for the interacting mutations as should be expected. This reflects the conditional nature of epistatic selection. We have included this analysis in the manuscript (starting on line 501).

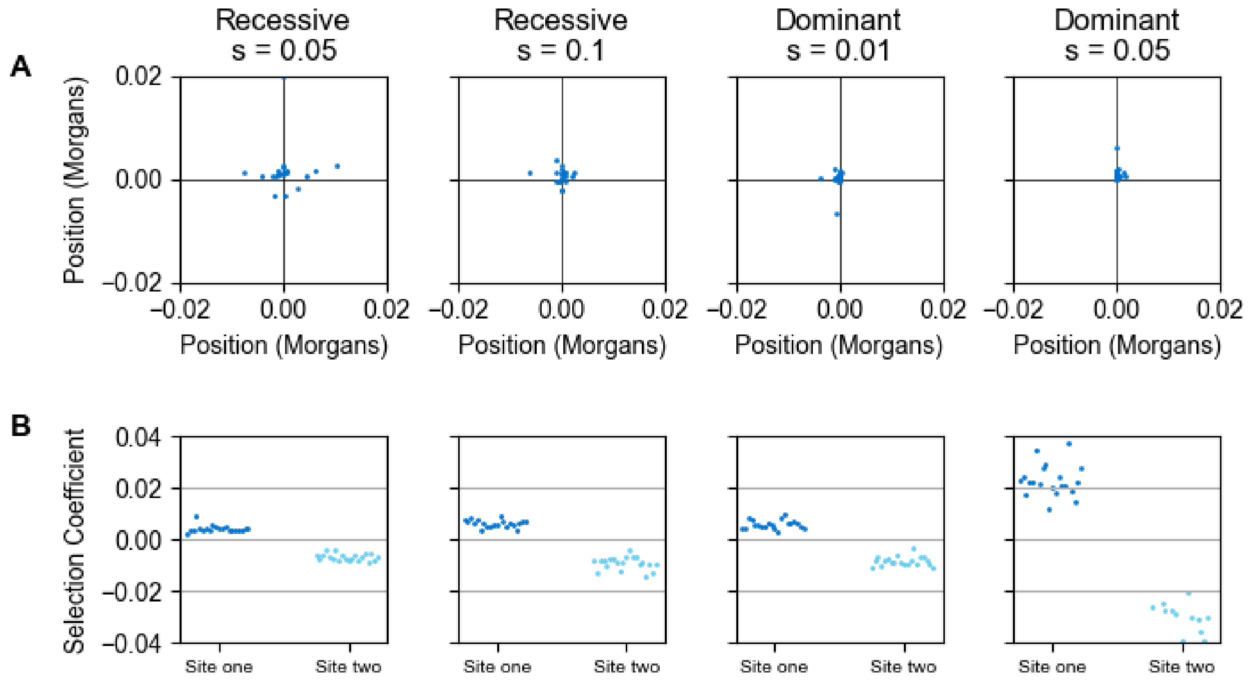

S8 Fig. Inference on epistatic loci. We simulated populations with two loci with a dominant or recessive epistatic interaction with varying selection coefficients (top of each column). Each population had an admixture fraction of 0.5 and was sampled 500 generations after admixture. (A) The position in Morgans of the two inferred selected sites (blue) and the simulated positions (black lines). The x-axis is the position of the first selected position, and the y-axis is the position of the second selected position. Each blue dot corresponds to fitting two sites on a single simulation. Both axes have been translated so that the simulated position is at 0 Morgans. (B) The inferred selection coefficients of the two sites (dark and light blue).

The documentation on github appears thorough and sufficient.

Thank you.

Minor comments

Line 93: Should give a bit more background on 'ancestry tracts'.

We've added some context on ancestry tracts.

Line 99: Phrasing is odd; reference to [43] comes later. 'Our' refers only to PI?

We've moved the reference up a sentence, and yes the 'our' refers to the PIs lab. We've made an adjustment to make this more clear.

Line 113: Consider highlighting upfront that 'multi-locus' selection is explicitly non-epistatic as used by the authors.

We've added a mention that the selection is non-epistatic in our method (line 81).

Line 132: I know that other approaches consider this, too (e.g., Wavelet transform out of Graham Coop's group). It would be nice to see a reference to a system or two where this could vs. could not be used. E.g., would it in flies but not baboons? Would it work in hybrid zones (where ancestry is clinal) or not?

We've run a simulation of several clinal populations sampled at varying generations since the start of the hybridization. We've found that on these populations our method can still discern between single and two site selection, once enough time has passed since the start of hybridization. The locations of the selected sites are accurately inferred once enough time has passed, but the selection coefficients appear to be underestimated presumably because migration introduces lower fitness alleles thereby decreasing the allele frequency change we would otherwise observe.

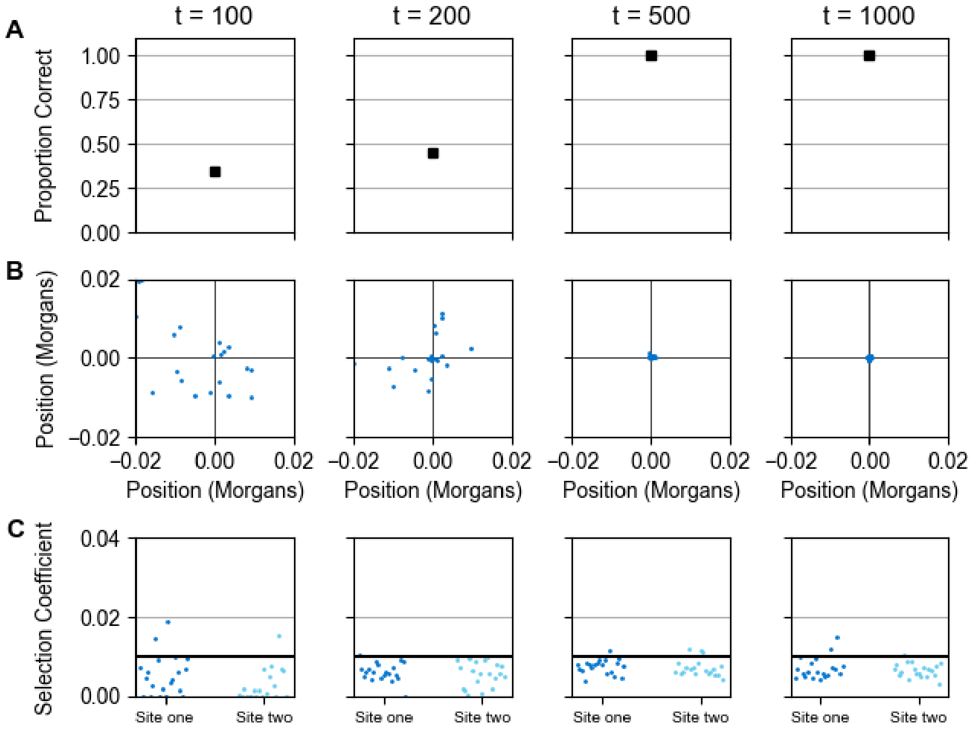

S7 Fig. Estimating parameters of selection on clinal hybrids. We simulated two-locus selection acting on hybrids in a cline, and used our method to distinguish between two-locus and single-locus selection. In each column, we vary the number of generations since initial hybridization after which we sample the population. These values are found at the top of each column. (A) The proportion of two site simulations in which the number of sites was correctly estimated. (B) The position in Morgans of the two inferred selected sites (blue) and the simulated positions (black lines). The x-axis is the position of the first selected position, and the y-axis is the position of the second selected position. Each blue dot corresponds to fitting two sites on a single simulation. Both axes have been translated so that the simulated position is at 0 Morgans. (C) The inferred selection coefficients of the two sites (dark and light blue), and the simulated selection coefficients (black).

These analyses have been added to the manuscript (starting line 482).

Line 158: It would be nice to see a similar approach investigating epistasis (line 166) / recurrent immigration.

We've now included investigations into epistatic interactions. Please see our response above.

We've also performed some simple simulations of admixed populations with recurrent migration from their ancestral populations to explore the effect that this migration has on our method. We simulated admixed populations with admixture parameters (m=0.2, t=500), which had two nearby selected positions, both with s = 0.01 . We also simulated null populations which only carried a single selected position. We tested our methods' ability to distinguish between these two cases, even in the presence of recurrent migration from the ancestral populations. We found that our method can distinguish between these two cases as long as the migration rate is around 0.0005 per generation or lower. We've included these analyses in the paper (line 482).

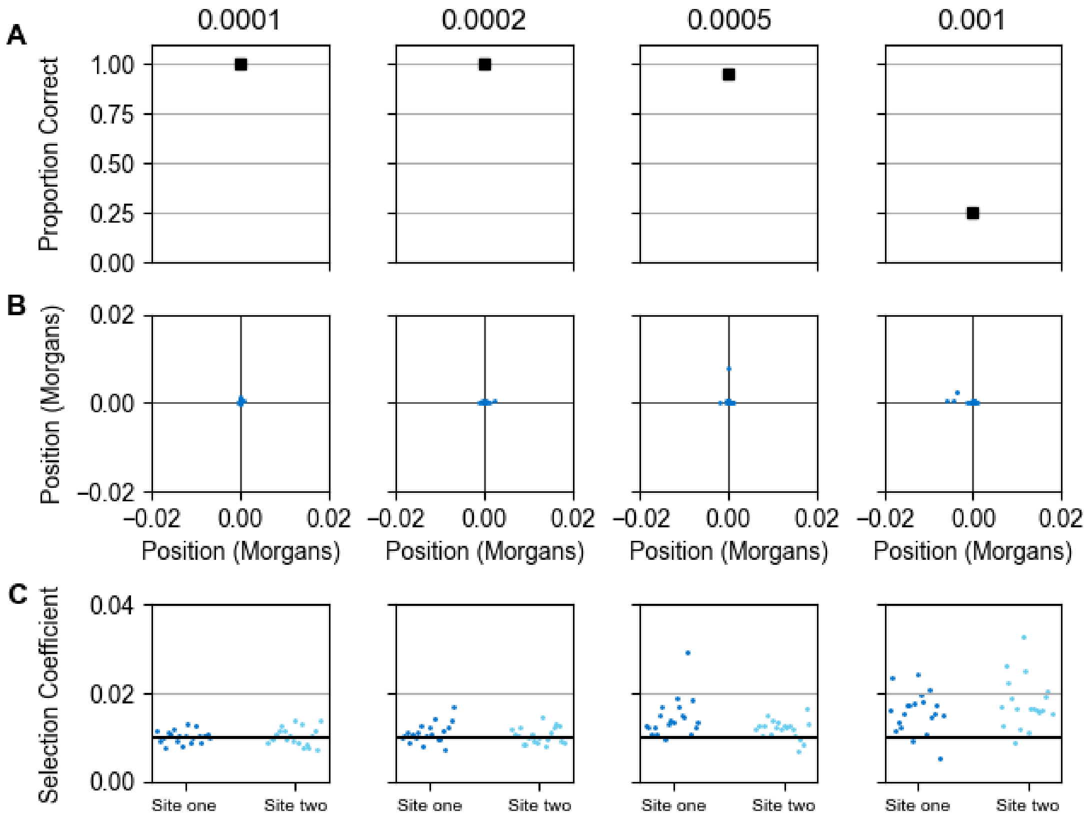

S6 Fig. Effects of recurrent migration. We simulated two-locus selection in populations which received recurrent migration from the ancestral populations at varying rates per generation (top of each column). Each population had an admixture fraction of 0.2 and was sampled 500 generations after admixture. (A) The proportion of two site simulations in which the number of sites was correctly estimated. (B) The position in Morgans of the two inferred selected sites (blue) and the simulated positions (black lines). The x-axis is the position of the first selected position, and the y-axis is the position of the second selected position. Each blue dot corresponds to fitting two sites on a single simulation. Both axes have been translated so that the simulated position is at 0 Morgans. (C) The inferred selection coefficients of the two sites (dark and light blue), and the simulated selection coefficients (black).

Line 241: Agree that this is problematic, but in recently admixed populations wouldn't such tightly linked sites essentially behave as a single locus?

This is basically correct. Within some small distance there will be none (or very few) recombination events that decouple local ancestry. Only after several generations will enough recombination events occur between the sites to make their individual effects apparent.

Line 295: Should probably note for non-fly readers what 3R vs 3L are, and highlight that these are different (some people might see '3' and think all '3' are created equally).

We've added a quick explanation on these components of the drosophila genome nomenclature.

Line 304: Unclear if you are comparing the two methods or what is known from non-HMM sequencing effort and geography/human history?

This is just comparing the slightly different demographic parameters you get when you infer them using genotype data from arm 3R vs 3L. We've added a small addition in the text to make this more clear.

Line 372: Can you explain a bit more about why 130K different site combinations is intractable? Is it the compute time for each site?

Yes, it takes some time to optimize the parameters of a single model (*e.g.,* approximately 10 minutes for a three selected site model). From that we can see that optimizing 130k models would be intractable. We have added a bit more context to this section.

Line 542: I think caveats like this should be brought to the main text. I also think that some reporting of Ne for empirical systems that people study (neanderthal/human, swordtails, some hybrid zones, flies, etc.) would put these caveats in better context.

We've further investigated the population size at which our method starts to break down, and found that it can still work with an effective population size of about 2000. We've put this into the main text (line 704), and brought it into context with some examples (line 424). Of course, these are guidelines and we recommend that users perform simulations under the null to evaluate the power for their specific system, sequencing effort, and other considerations.

Fig 1: This is an excellent figure! Great job to the authors, it really makes a complicated iterative process clear. (i) Giving a legend indicating what colours represent in the figure itself (for ancestry proportion and also lnL ratios) would be helpful.

Thanks for the feedback and we're glad the reviewer found this helpful! We have added a legend to make the figure clearer.

Fig 2: Using 'm' as minor parent ancestry fraction was initially confusing to me. I thought it was originally migration. Is this convention in the field? Also, these success rates seem quite worrying for recently admixed populations—are the authors concerned about this at all? What is the age of most populations that people are studying?

Yes, 'm' is commonly used to refer to the minor parent ancestry fraction, or the admixture proportion. We have taken care to avoid confusion by using explicit terms rather than the letter 'm' when we discuss recurrent migration. The text has been modified accordingly. We have also made the consideration of population age more explicit in the caveat section.

Figure 3: Would seem that site 1 and site 2 are redundant for the two site, so why not just show the mean and simplify the figure?

We believe it's important to show both inferred selection coefficients, to show that both sites on their own are being accurately inferred. If we only reported the mean, there could be a case where in a simulation of two sites with s = 0.01, our method incorrectly infers that one site had s=0.02, and the other site had a nearly zero selection coefficient.

Figure 4: Great figure. Performance is quite good.

Thanks.

Reviewer #3: Summary

Previous methods to identify sites under selection do not take into account linkage between such sites, which can be especially pronounced in admixed populations in which multiple adaptive loci are introduced simultaneously. To address this, the authors develop a tool (AHMM-MLS) to identify multiple linked sites under selection in an admixed population. The method also allows for the estimation of the strength of selection of the linked adaptive sites. The authors apply AHMM-MLS to a population of D. melanogaster and are able to identify linked sites under selection. They find that when the effects of multiple selected sites are not considered the selection coefficient might be overestimated.

My major comments are below:

- It seems that recombination is an important parameter for being able to discern whether 1, 2, 3, or 4 adaptive mutations are present. For example, the authors write "We found that one and two site selection models are easier to distinguish as t increases, and as the distance between the sites increases" and "In most cases, our method performed better as the time since admixture increased". It seems that recombination unlinking sites that were introgressed on the same haplotype is key – could the authors test their approach as a function of rho? Additionally, a sentence or two explaining the intution behind why the method performs better as a time since admixture increases would be helpful.

Please see our response to reviewers 1 and 2 above who raised similar concerns about the impact of recombination misspecification on the performance of our method.

Additionally, we have added a sentence to the manuscript explaining why more time since admixture improves the methods (line 342). Briefly, when selection coefficients are modest, it requires some time for the allele frequencies at selected sites and linked positions to change. More time allows selected sites to differentiate from the background rate of introgression.

- However, this model seems to be at odds with more recent introgression events involving short segments. More broadly, the selection of 1, 2, 3,4 mutations seems artificial. For example, in a paper by Birzu et al. 2023 BioRxiv (https://www.biorxiv.org/content/10.1101/2023.06.06.543983.abstract), it seems that numerous loci (O(100)) are introduced in adaptive introgressions of chunks of short loci (e.g. 200bps) bearing beneficial mutations rather than Mb long regions like those explored in this paper. Would this method miss such events? And, why is it important to estimate the number of loci under selection? Could we not just have an inference of 1 vs >1? That in and of itself could be interesting to know whether polygenic seleciton is acting or not.

We thank the reviewer for this observation. The paper they provide describes gene transfer in a bacterial population wherein recombination may be substantially different than in obligate sexual populations. Our method is explicitly designed to estimate the selection and introgression landscape within admixed sexually recombining eukaryotic populations. For this

reason it would not be appropriate for the case described here. We have now added clarification in the main text describing which types of populations our approach will be suitable for (line 133).

The core innovation of our work is that by modeling the process of recombination and selection we can accurately identify and estimate the selection coefficients even when sites are closely linked as is sometimes the case for admixed populations. One can certainly use our method to determine whether there is more than one selected locus and therefore polygenic adaptation is supported as suggested by the reviewer.

- In Fig 3 I don't understand if for the single site model you test for a single site versus neutral at each site independently or just the site with highest likelihood? Would the higher selection strength on the one site model be the selection strength for both sites?

For the single site we test the locus with the highest likelihood, and we do the same for the two-site and the multi-site models. We found that the higher selection strength on the one site model does not correspond to the sum of the selection strengths of both sites, it is usually a bit less.

- In Fig 4 why was an admixture time of 1000 generations not shown (as in Fig 3) ?

We've added in additional simulations at 100 generations and 1000 generations, as in the previous figures.

- For the demographic model inference the authors use chromosome 3L as they did not find evidence of alleles under positive selection on that chromosome in a previous study. Chromosome 3L may not show evidence of being under positive selection but that does not mean it is neutral and good for demographic estimation. There could be BGS, or weaker positive selection that could potentially confound the demographic inference. Were additional controls included to account for this? Also it is unclear what the demographic model inferred is? Is it only the admixture fraction and time or do the authors estimate more demographic parameters? If so, could the authors report these?

We do not estimate additional parameters beyond the admixture time and admixture fraction. We note that our previous work (Medina et al. 2018), analyzed the demographic history of this population in detail and found a single pulse admixture model was the best fit to the data. Furthermore, we note that the demographic inference method is robust to a large range of weak selection effects (Corbett-Detig and Nielsen 2016) and we now describe this as well as the caveat that additional forms of selection are not considered in the main text (line 545).

- On chromosomes 2R and 3R there are some well documented positive controls (for instance at Ace, CHKoV1, and Cyp6g1). Can the authors recover the signal from any known positive controls?

On chromosome 3R we can recover a strong selection signal at the Ace locus, it's one of the sites with the strongest selection coefficients in our multi-locus model. Our method overlooks the CHKoV1 locus on 3R, which was a problem that we encountered on this system with our previous method as well. We believe that we can't recover a signal at this locus because the variants responsible for infection resistance were thought to be present in both ancestral populations. We have added this information to the manuscript (line 578).

- When applying AHMM-MLS to Drosophila (lines 323-334), what multi locus model are the authors testing: only up to two sites, or up to four (which is the most they tested in results previously) or more? If they are testing >4 sites, it is unclear to me what happens with the sites that were discarded while iterating through all sites. Do they start again with the sites that were discarded? Or do all ~9 sites have to be in linkage? Or

is the method able to identify all sites under selection even if they are not linked?

We apologize for the confusion, when applying the iterative method to Drosophila we test models with up to 10 sites. At some point we reach 9 sites in our accepted model, and when we test the remaining sites we include the 9 previously accepted sites. When a site is discarded, we do not add it to our accepted model, and we simply test the next site against our accepted model. Our method is able to simultaneously infer the selection coefficients of multiple sites even if they are not linked.

- Some figures referred to throughout the methods could be referenced in the main results section. Particularly Fig S9 could be referenced somewhere in lines 158-160.

We've added the reference.

- In lines 158-160: "Below we evaluate the impacts of population size …" , could the authors specify that they do this in the methods section.

We've clarified this section.

- Line 568: "selection coefficients are not expected to change substantially" Did the authors test for Ne misspecification? Can the authors show that selection strength is not impacted too much when Ne is misspecified. (I believe the authors only look at what happens when time since admixture or admixture proportion is misspecified).

We have now done some analysis on the effect of Ne misspecification, and found that for larger population sizes there is very little effect. We expect that inferring a smaller population size than the true value will make our method more conservative in the selection of sites. Our comment related to the selection coefficients was referring to the potential inclusion of additional selected positions in our model if our method was less conservative. We believe that the selection coefficients would not change substantially because we believe we have captured the large effect loci throughout the chromosome, and additional sites would have a weaker impact on the model.

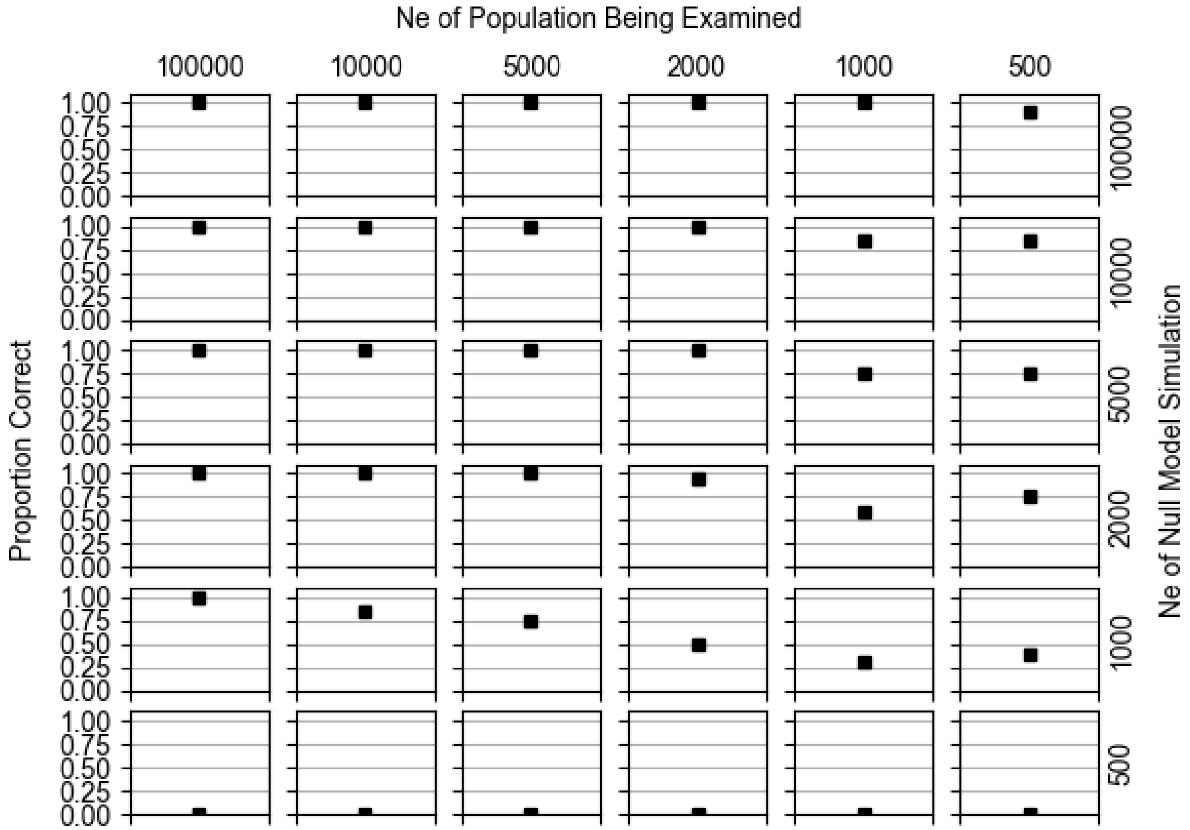

Effect of Ne misspecification on null model simulations. For each column we simulated 20 populations with our standard demographic and selection parameters (t = 500, m = 0.2, two sites with s = 0.01 with a distance of 2 centimorgans) of varying sizes. For each row we simulated 20 null model populations with the same demographic and selection parameters except with only a single site of selection. Each graph shows the proportion of two site simulations whose lnL ratio surpassed 95% of the lnL ratios of the corresponding null model simulations.

Minor Comments

- Line 66: Linkages → linkage? **We've removed the s**
- Line 121: Add citation to " our previous method". **Done**
- Figure 1. Legend for null and alternative models would be helpful, i.e orange = null , blue = alternative.

We've added a legend to this figure, and we agree that this makes it clearer.

- Fig 2. Title for axis on the right "distance between selected positions"

We've added additional titles to make the figure clearer.

- Line 212: Add units to " t from 100 to 1000" (generations?)

We've made the change

- Fig 4. Specify what admixture proportion you're using here.

We've added that information.
- Line 512: Did you have 20 simulations per each scenario? If so, that seems quite low.

We did. This is because we evaluated many distinct scenarios and including a full forward simulation of each can be quite resource consuming. Although 20 simulations may not always provide a precise point estimate of accuracy for a given set of parameters, the broad range of parameters that we evaluated allows us to identify the major trends.

Have all data underlying the figures and results presented in the manuscript been provided?
Large-scale datasets should be made available via a public repository as described in the *PLOS Genetics* data availability policy, and numerical data that underlies graphs or summary statistics should be provided in spreadsheet form as supporting information.

Reviewer #1: Yes

Reviewer #2: Yes

Reviewer #3: None

PLOS authors have the option to publish the peer review history of their article (what does this mean?). If published, this will include your full peer review and any attached files.

If you choose "no", your identity will remain anonymous but your review may still be made public.

Do you want your identity to be public for this peer review? For information about this choice, including consent withdrawal, please see our Privacy Policy.

Reviewer #1: No

Reviewer #2: No

Reviewer #3: No
