# Supplementary material for: Inferring multi-locus selection in admixed populations
Source: PLoS Genet. 2023 Nov 28;19(11):e1011062. doi: 10.1371/journal.pgen.1011062 (PMC10707604; doi:10.1371/journal.pgen.1011062)
Supplement: Peer review history

# Peer review: Decision Letter 0

**DOI:** 10.1371/journal.pgen.1011062.r001

**Authors:** Xiaofeng Zhu, Nicolas Bierne

25 Jul 2023

Dear Dr Ayala,

Thank you very much for submitting your Research Article entitled 'Inferring multi-locus selection in admixed populations' to PLOS Genetics.

The manuscript was fully evaluated at the editorial level and by independent peer reviewers. The reviewers appreciated the attention to an important problem, but raised some substantial concerns about the current manuscript. Based on the reviews, we will not be able to accept this version of the manuscript, but we would be willing to review a much-revised version. We cannot, of course, promise publication at that time.

Should you decide to revise the manuscript for further consideration here, your revisions should address the specific points made by each reviewer. We will also require a detailed list of your responses to the review comments and a description of the changes you have made in the manuscript.

If you decide to revise the manuscript for further consideration at PLOS Genetics, please aim to resubmit within the next 60 days, unless it will take extra time to address the concerns of the reviewers, in which case we would appreciate an expected resubmission date by email to plosgenetics@plos.org.

If present, accompanying reviewer attachments are included with this email; please notify the journal office if any appear to be missing. They will also be available for download from the link below. You can use this link to log into the system when you are ready to submit a revised version, having first consulted our Submission Checklist.

To enhance the reproducibility of your results, we recommend that you deposit your laboratory protocols in protocols.io, where a protocol can be assigned its own identifier (DOI) such that it can be cited independently in the future. Additionally, PLOS ONE offers an option to publish peer-reviewed clinical study protocols. Read more information on sharing protocols at https://plos.org/protocols?utm_medium=editorial-email&utm_source=authorletters&utm_campaign=protocols

Please be aware that our data availability policy requires that all numerical data underlying graphs or summary statistics are included with the submission, and you will need to provide this upon resubmission if not already present. In addition, we do not permit the inclusion of phrases such as "data not shown" or "unpublished results" in manuscripts. All points should be backed up by data provided with the submission.

While revising your submission, please upload your figure files to the Preflight Analysis and Conversion Engine (PACE) digital diagnostic tool.  PACE helps ensure that figures meet PLOS requirements. To use PACE, you must first register as a user. Then, login and navigate to the UPLOAD tab, where you will find detailed instructions on how to use the tool. If you encounter any issues or have any questions when using PACE, please email us at figures@plos.org.

PLOS has incorporated Similarity Check, powered by iThenticate, into its journal-wide submission system in order to screen submitted content for originality before publication. Each PLOS journal undertakes screening on a proportion of submitted articles. You will be contacted if needed following the screening process.

To resubmit, use the link below and 'Revise Submission' in the 'Submissions Needing Revision' folder.

We are sorry that we cannot be more positive about your manuscript at this stage. Please do not hesitate to contact us if you have any concerns or questions.

Yours sincerely,

Nicolas Bierne

Academic Editor

PLOS Genetics

Xiaofeng Zhu

Section Editor

PLOS Genetics

Dear Dr Ayala:

We have received three thoughtful reviews of your manuscript entitled "Inferring multi-locus selection in admixed populations". All three reviewers and I enjoyed the manuscript and agreed that, despite the limitations and caveats of the method, this is a valuable step towards better analysing adaptive introgression in admixed populations.

The reviewers did make a number of recommendations to investigate some of the confounding effects in more detail and to improve the clarity of the manuscript. Most importantly, they ask you to better explore and discuss the effects of recombination and demography (including when they are misinfered) and the neglect of epistasis. Although you have done a good job of evaluating the performance of the method, some questions remain about its applicability to real data in a general context, e.g. what if the recombination map or demography is incorrect or biased (reviewers 1 and 3)? In admixed populations, there is often contrasting selection between different genomic regions, with genome-wide purging of the minor parent ancestry when ancestry variance is high in the first few generations after the admixture pulse. Negative and epistatic selection can make the admixture rate (m) different between genomic regions (with different recombination rates or density of selected genes), and this can be a problem unless the method is robust to deviations from the true local m on the chromosomal stretch under study. Adaptive introgression can sometimes be seen more as a resistance to this purging erosion, and this can produce the multi-peak pattern you are interested in (referee 2).

Finally, you should include some more details directly in the manuscript that we need to find in your previous work. Ancestry_HMM-S has been published recently and you should be more explicit about the difference between the two methods (not just that AHMM-S is mono-locus and AHMM-MLS is multi-locus): your numerical method implies inferring t and m from genomic regions assumed to be neutral (and this will need to be better adressed in the revised version), whereas, as far as I understand, the Shchur et al. approximations allow all-in-1 inference in AHMM-S. Could you also clarify what you mean by ancestry informative positions? The Drosophila example should also be better clarified. We want to know what the other chromosomes look like, and we need to read previous manuscripts to get that information. The data should be accessible and perhaps some supporting figures should be provided to describe the system, e.g. population differentiation, linkage disequilibrium blocks, Marey maps, etc. In Svedberg et al. selection for resistance was discussed at length, whereas it is missing here (reviewer 3). Still, it remains puzzling why resistance genes are clustered on chromosome 3R (and each have s~0.01 with admixture proportions ~0.6) while some other chromosome arms are free from selection for resistance genes. In the end, it looks like you developed AHMM-MLS to improve the inference made on this system with AHMM-S, and this does not show that your method is of interest in other systems where a few adaptive introgression breakthroughs are observed, or where positive, negative and epistatic selection is all over the genome. It would be more impactful to apply it to another system.

Please try to address all of the referees' concerns in a revised version. I'm looking forward to reading it.

Best regards,

Nicolas Bierne

Reviewer's Responses to Questions

**Comments to the Authors:**

**Please note here if the review is uploaded as an attachment.**

Reviewer #1: The manuscript by Ayala and Corbett-Detig presents a HMM method to infer the strength of location acting on admixed ancestry that can be applicable to genome-wide data. One important advance in their method is to model natural selection at multiple sites simultaneously. Through simulations, they show that if in truth multiple sites are experiencing selection, but a model with only one site under selection is fit to the data, the strength of selection will be overestimated. Thus, modeling multiple sites under selection is important for accurate inference.

Overall, I really enjoyed this manuscript. I think the method is quite clever and innovative. It also addresses a significant research question—how to best infer selection on alleles recently brought into a new population due to admixture. The authors have done a nice job evaluating the performance of their method under different circumstances using simulations and show that it performs well. Applying their method to Drosophila data, the authors found 9 selected sites on chromosome arm 3R and that the selection coefficients in previous work were likely overestimated due to the cryptic presence of multiple linked SNPs experiencing selection.

While I think the authors did a nice job on testing robustness to violations of the model and mis-specifying certain parameters, one area where I think more work is needed relates to the recombination rate in the region of interest. In particular, based on my understanding, it seems that both selection and recombination affect and change haplotype frequencies in the model. As such, accurate estimates of the recombination rate must be needed. This raises several questions that the authors should try to address. First, how do the authors recommend that uncertainty in the recombination rate be dealt with when using their method? Second, I think the authors should undertake additional simulations where the true recombination rate used to generate the data is different from the rate assumed in the inference. How much does performance change in this situation? In particular, is the false-positive rate elevated when the wrong recombination rate is assumed in the inference? Is the location of the selected site(s) mis-inferred in this situation? I think more exploration of recombination is necessary.

I also have a few more minor comments:

1) Line 145-146: “Forward equations” are mentioned. I assume that the authors are referring to the forward algorithm to calculate the probability of the data under the model for HMMs. Maybe specify this and provide a reference here for the uninitiated reader.

2) I really like Figure 1!

3) Methods, haplotype frequency matrix: I think a cartoon figure of the H matrix might make the presentation a bit clearer.

4) Methods, While the authors do a nice job explaining different parts and components of their method, I think some additional explanation about how the method would be applied to genome-wide data would be beneficial. For example, is the same M matrix used for all sites across the genome? Or, are different Ms computed? How does this vary with recombination rate? Is M recomputed for each s and h value?

Reviewer #2: In this manuscript, Ayala & Corbett-Detig present a new approach—an extention of the PI’s previous work—to detect multi-locus selection along chromosomes. The main advance here is that the approach accounts for linkage between selected sites to identify them an estimate their selection coefficients. The approach has many limitations and caveats that are duly acknowledged by the authors, for example not accounting for epistasis (ubiquitous in hybrids), assuming a single admixture pulse, and performs poorly in recent hybrid populations (few gens of admixture), at estimating dominance, and with small-effect alleles. However, the authors also show clearly that existing approaches are likely to greatly overestimate selection. This approach is clearly a step forward for the field.

The only major comment that I have is that I’d like to see a bit more investigation into epistasis. As noted above, the major limiting assumption of the work is that it does not consider epistasis, which is likely rampant in admixed populations. However, this is a common assumption in some key theoretical models about selection in admixed populations (e.g., Veller et al. Evolution 2023) so should by no means preclude publication. However, as the goal is for this approach to be applied to real datasets, I would like to see a brief foray into some very simple epistasis in the validation stage. If there are recessive DMIs, for example, what do the outputs of the model look like?

The documentation on github appears thorough and sufficient.

Minor comments

Line 93: Should give a bit more background on ‘ancestry tracts’.

Line 99: Phrasing is odd; reference to [43] comes later. ‘Our’ refers only to PI?

Line 113: Consider highlighting upfront that ‘multi-locus’ selection is explicitly non-epistatic as used by the authors.

Line 132: I know that other approaches consider this, too (e.g., Wavelet transform out of Graham Coop’s group). It would be nice to see a reference to a system or two where this could vs. could not be used. E.g., would it in flies but not baboons? Would it work in hybrid zones (where ancestry is clinal) or not?

Line 158: It would be nice to see a similar approach investigating epistasis (line 166) / recurrent immigration.

Line 241: Agree that this is problematic, but in recently admixed populations wouldn’t such tightly linked sites essentially behave as a single locus?

Line 295: Should probably note for non-fly readers what 3R vs 3L are, and highlight that these are different (some people might see ‘3’ and think all ‘3’ are created equally).

Line 304: Unclear if you are comparing the two methods or what is known from non-HMM sequencing effort and geography/human history?

Line 372: Can you explain a bit more about why 130K different site combinations is intractable? Is it the compute time for each site?

Line 542: I think caveats like this should be brought to the main text. I also think that some reporting of Ne for empirical systems that people study (neanderthal/human, swordtails, some hybrid zones, flies, etc.) would put these caveats in better context.

Fig 1: This is an excellent figure! Great job to the authors, it really makes a complicated iterative process clear. (i) Giving a legend indicating what colours represent in the figure itself (for ancestry proportion and also lnL ratios) would be helpful.

Fig 2: Using ‘m’ as minor parent ancestry fraction was initially confusing to me. I thought it was originally migration. Is this convention in the field? Also, these success rates seem quite worrying for recently admixed populations—are the authors concerned about this at all? What is the age of most populations that people are studying?

Figure 3: Would seem that site 1 and site 2 are redundant for the two site, so why not just show the mean and simplify the figure?

Figure 4: Great figure. Performance is quite good.

Reviewer #3: Summary

Previous methods to identify sites under selection do not take into account linkage between such sites, which can be especially pronounced in admixed populations in which multiple adaptive loci are introduced simultaneously. To address this, the authors develop a tool (AHMM-MLS) to identify multiple linked sites under selection in an admixed population. The method also allows for the estimation of the strength of selection of the linked adaptive sites. The authors apply AHMM-MLS to a population of D. melanogaster and are able to identify linked sites under selection. They find that when the effects of multiple selected sites are not considered the selection coefficient might be overestimated.

My major comments are below:

- It seems that recombination is an important parameter for being able to discern whether 1, 2, 3, or 4 adaptive mutations are present. For example, the authors write “We found that one and two site selection models are easier to distinguish as t increases, and as the distance between the sites increases” and “In most cases, our method performed better as the time since admixture increased”. It seems that recombination unlinking sites that were introgressed on the same haplotype is key – could the authors test their approach as a function of rho? Additionally, a sentence or two explaining the intution behind why the method performs better as a time since admixture increases would be helpful.

- However, this model seems to be at odds with more recent introgression events involving short segments. More broadly, the selection of 1, 2, 3,4 mutations seems artificial. For example, in a paper by Birzu et al. 2023 BioRxiv (https://www.biorxiv.org/content/10.1101/2023.06.06.543983.abstract), it seems that numerous loci (O(100)) are introduced in adaptive introgressions of chunks of short loci (e.g. 200bps) bearing beneficial mutations rather than Mb long regions like those explored in this paper. Would this method miss such events? And, why is it important to estimate the number of loci under selection? Could we not just have an inference of 1 vs >1? That in and of itself could be interesting to know whether polygenic seleciton is acting or not.

- In Fig 3 I don’t understand if for the single site model you test for a single site versus neutral at each site independently or just the site with highest likelihood? Would the higher selection strength on the one site model be the selection strength for both sites?

- In Fig 4 why was an admixture time of 1000 generations not shown (as in Fig 3) ?

- For the demographic model inference the authors use chromosome 3L as they did not find evidence of alleles under positive selection on that chromosome in a previous study. Chromosome 3L may not show evidence of being under positive selection but that does not mean it is neutral and good for demographic estimation. There could be BGS, or weaker positive selection that could potentially confound the demographic inference. Were additional controls included to account for this? Also it is unclear what the demographic model inferred is? Is it only the admixture fraction and time or do the authors estimate more demographic parameters? If so, could the authors report these?

- On chromosomes 2R and 3R there are some well documented positive controls (for instance at Ace, CHKoV1, and Cyp6g1). Can the authors recover the signal from any known positive controls?

- When applying AHMM-MLS to Drosophila (lines 323-334), what multi locus model are the authors testing: only up to two sites, or up to four (which is the most they tested in results previously) or more? If they are testing >4 sites, it is unclear to me what happens with the sites that were discarded while iterating through all sites. Do they start again with the sites that were discarded? Or do all ~9 sites have to be in linkage? Or is the method able to identify all sites under selection even if they are not linked?

- Some figures referred to throughout the methods could be referenced in the main results section. Particularly Fig S9 could be referenced somewhere in lines 158-160.

- In lines 158-160: “Below we evaluate the impacts of population size …” , could the authors specify that they do this in the methods section.

- Line 568: “selection coefficients are not expected to change substantially” Did the authors test for Ne misspecification? Can the authors show that selection strength is not impacted too much when Ne is misspecified. ( I believe the authors only look at what happens when time since admixture or admixture proportion is misspecified).

Minor Comments

- Line 66: Linkages → linkage?

- Line 121: Add citation to “ our previous method”.

- Figure 1. Legend for null and alternative models would be helpful, i.e orange = null , blue = alternative.

- Fig 2. Title for axis on the right “distance between selected positions”

- Line 212: Add units to “ t from 100 to 1000” (generations?)

- Fig 4. Specify what admixture proportion you’re using here.

- Line 512: Did you have 20 simulations per each scenario? If so, that seems quite low.

**********

**Have all data underlying the figures and results presented in the manuscript been provided?**

Large-scale datasets should be made available via a public repository as described in the *PLOS Genetics* data availability policy, and numerical data that underlies graphs or summary statistics should be provided in spreadsheet form as supporting information.

Reviewer #1: Yes

Reviewer #2: Yes

Reviewer #3: None

**********

PLOS authors have the option to publish the peer review history of their article (what does this mean?). If published, this will include your full peer review and any attached files.

If you choose “no”, your identity will remain anonymous but your review may still be made public.

**Do you want your identity to be public for this peer review?** For information about this choice, including consent withdrawal, please see our Privacy Policy.

Reviewer #1: No

Reviewer #2: No

Reviewer #3: No

# Author comment: Author response to Decision Letter 0

**DOI:** 10.1371/journal.pgen.1011062.r002

23 Oct 2023

Attachment Submitted filename: Revision Response.pdf Click here for additional data file.

# Peer review: Decision Letter 1

**DOI:** 10.1371/journal.pgen.1011062.r003

**Authors:** Xiaofeng Zhu, Nicolas Bierne

13 Nov 2023

Dear Dr Ayala,

We are pleased to inform you that your manuscript entitled "Inferring multi-locus selection in admixed populations" has been editorially accepted for publication in PLOS Genetics. Congratulations!

Before your submission can be formally accepted and sent to production you will need to complete our formatting changes, which you will receive in a follow up email. Please be aware that it may take several days for you to receive this email; during this time no action is required by you. Please note: the accept date on your published article will reflect the date of this provisional acceptance, but your manuscript will not be scheduled for publication until the required changes have been made.

Once your paper is formally accepted, an uncorrected proof of your manuscript will be published online ahead of the final version, unless you’ve already opted out via the online submission form. If, for any reason, you do not want an earlier version of your manuscript published online or are unsure if you have already indicated as such, please let the journal staff know immediately at plosgenetics@plos.org.

In the meantime, please log into Editorial Manager at https://www.editorialmanager.com/pgenetics/, click the "Update My Information" link at the top of the page, and update your user information to ensure an efficient production and billing process. Note that PLOS requires an ORCID iD for all corresponding authors. Therefore, please ensure that you have an ORCID iD and that it is validated in Editorial Manager. To do this, go to ‘Update my Information’ (in the upper left-hand corner of the main menu), and click on the Fetch/Validate link next to the ORCID field.  This will take you to the ORCID site and allow you to create a new iD or authenticate a pre-existing iD in Editorial Manager.

If you have a press-related query, or would like to know about making your underlying data available (as you will be aware, this is required for publication), please see the end of this email. If your institution or institutions have a press office, please notify them about your upcoming article at this point, to enable them to help maximise its impact. Inform journal staff as soon as possible if you are preparing a press release for your article and need a publication date.

Thank you again for supporting open-access publishing; we are looking forward to publishing your work in PLOS Genetics!

Yours sincerely,

Nicolas Bierne

Academic Editor

PLOS Genetics

Xiaofeng Zhu

Section Editor

PLOS Genetics

www.plosgenetics.org

Twitter: @PLOSGenetics

----------------------------------------------------

Comments from the reviewers (if applicable):

Dear Dr Ayala, all three referees are satisfied with your revisions and I am therefore delighted to accept your manuscript for publication in PLOS Genetics.

Best regards,

Nicolas Bierne

Reviewer's Responses to Questions

**Comments to the Authors:**

**Please note here if the review is uploaded as an attachment.**

Reviewer #1: The authors have significantly improved the manuscript and have satisfactorily addressed all of my previous concerns.

Reviewer #2: Great job by the authors here! I really appreciate how you went above and beyond to engage with the comments.

Reviewer #3: Thank you for addressing our comments.

**********

**Have all data underlying the figures and results presented in the manuscript been provided?**

Large-scale datasets should be made available via a public repository as described in the *PLOS Genetics* data availability policy, and numerical data that underlies graphs or summary statistics should be provided in spreadsheet form as supporting information.

Reviewer #1: Yes

Reviewer #2: Yes

Reviewer #3: Yes

**********

PLOS authors have the option to publish the peer review history of their article (what does this mean?). If published, this will include your full peer review and any attached files.

If you choose “no”, your identity will remain anonymous but your review may still be made public.

**Do you want your identity to be public for this peer review?** For information about this choice, including consent withdrawal, please see our Privacy Policy.

Reviewer #1: No

Reviewer #2: No

Reviewer #3: No

----------------------------------------------------

**Data Deposition**

If you have submitted a Research Article or Front Matter that has associated data that are not suitable for deposition in a subject-specific public repository (such as GenBank or ArrayExpress), one way to make that data available is to deposit it in the Dryad Digital Repository. As you may recall, we ask all authors to agree to make data available; this is one way to achieve that. A full list of recommended repositories can be found on our website.

The following link will take you to the Dryad record for your article, so you won't have to re‐enter its bibliographic information, and can upload your files directly: 

http://datadryad.org/submit?journalID=pgenetics&manu=PGENETICS-D-23-00578R1

More information about depositing data in Dryad is available at http://www.datadryad.org/depositing. If you experience any difficulties in submitting your data, please contact help@datadryad.org for support.

Additionally, please be aware that our data availability policy requires that all numerical data underlying display items are included with the submission, and you will need to provide this before we can formally accept your manuscript, if not already present.

----------------------------------------------------

**Press Queries**

If you or your institution will be preparing press materials for this manuscript, or if you need to know your paper's publication date for media purposes, please inform the journal staff as soon as possible so that your submission can be scheduled accordingly. Your manuscript will remain under a strict press embargo until the publication date and time. This means an early version of your manuscript will not be published ahead of your final version. PLOS Genetics may also choose to issue a press release for your article. If there's anything the journal should know or you'd like more information, please get in touch via plosgenetics@plos.org.

# Editor report: Acceptance letter

**DOI:** 10.1371/journal.pgen.1011062.r004

**Authors:** Xiaofeng Zhu, Nicolas Bierne

23 Nov 2023

PGENETICS-D-23-00578R1 

Inferring multi-locus selection in admixed populations 

Dear Dr Ayala, 

We are pleased to inform you that your manuscript entitled "Inferring multi-locus selection in admixed populations" has been formally accepted for publication in PLOS Genetics! Your manuscript is now with our production department and you will be notified of the publication date in due course.

The corresponding author will soon be receiving a typeset proof for review, to ensure errors have not been introduced during production. Please review the PDF proof of your manuscript carefully, as this is the last chance to correct any errors. Please note that major changes, or those which affect the scientific understanding of the work, will likely cause delays to the publication date of your manuscript. 

Soon after your final files are uploaded, unless you have opted out or your manuscript is a front-matter piece, the early version of your manuscript will be published online. The date of the early version will be your article's publication date. The final article will be published to the same URL, and all versions of the paper will be accessible to readers.

Thank you again for supporting PLOS Genetics and open-access publishing. We are looking forward to publishing your work! 

With kind regards,

Anita Estes

PLOS Genetics

On behalf of:

The PLOS Genetics Team

Carlyle House, Carlyle Road, Cambridge CB4 3DN | United Kingdom

plosgenetics@plos.org | +44 (0) 1223-442823

plosgenetics.org | Twitter: @PLOSGenetics
